# Identifying factors associated with instructor implementation of three-dimensional assessment in undergraduate biology courses

**DOI:** 10.1371/journal.pone.0312252

**Published:** 2024-10-22

**Authors:** Crystal Uminski, Brian A. Couch

**Affiliations:** 1 School of Biological Sciences, University of Nebraska, Lincoln, Nebraska, United States of America; 2 Thomas H. Gosnell School of Life Sciences, Rochester Institute of Technology, Rochester, New York, United States of America; Florida Agricultural and Mechanical University, UNITED STATES OF AMERICA

## Abstract

Recent national calls to transform undergraduate science education have centered on engaging students in scientific practices as a means to help them develop deeper insights into science. The three-dimensional framework for science education encapsulates the goals of these national calls by recommending that instructors integrate scientific practices, crosscutting concepts, and disciplinary core ideas throughout their courses. Prior research has found that introductory undergraduate biology exams contain few three-dimensional items suggesting that instructors likely face barriers in meeting the goals of national calls. To better understand these putative challenges, we explored factors potentially associated with three-dimensional alignment. Our generalized linear mixed model indicated that instructors who used three-dimensional items on their exams were more likely to use Bloom’s Taxonomy when designing their exams and were more likely to write these items using a constructed-response format. We also found that professional development opportunities did not necessarily change the likelihood an instructor would employ three-dimensional items. We previously identified that few items in our sample fully aligned to scientific practices, making scientific practices the limiting dimension for three-dimensional alignment. Our subsequent analysis here revealed that most biology instructors had exam items that were at least partially aligned to scientific practices. Based on our results, we highlight the significant time and resources that instructors likely need to write and grade constructed-response assessments, suggest that instructors build on items that are mostly aligned to scientific practices to increase their three-dimensional alignment, and propose ways that professional development programs and communities might further support instructors in meeting national calls.

## Introduction

For the past several decades, the landscape of science education has been defined by national calls for teaching that engages students in scientific processes to help them better understand science [1–12]. These calls have spotlighted particular aspects of science education, such as scientific literacy [1], inquiry [9, 13], career preparation [5, 8, 11], and integrating scientific concepts and competencies [4, 12]. Within the K-12 education system, public school districts are often held accountable for achieving the goals outlined in these calls through standardized assessments, accountability-based policies, and federal intervention programs [14, 15]; however, postsecondary education lacks equivalent structures to monitor progress [16]. Thus, the extent to which national calls have percolated through the undergraduate biology education system remains an area of active research.

### Gauging implementation of three-dimensional learning through course exams

**Fig 1:**
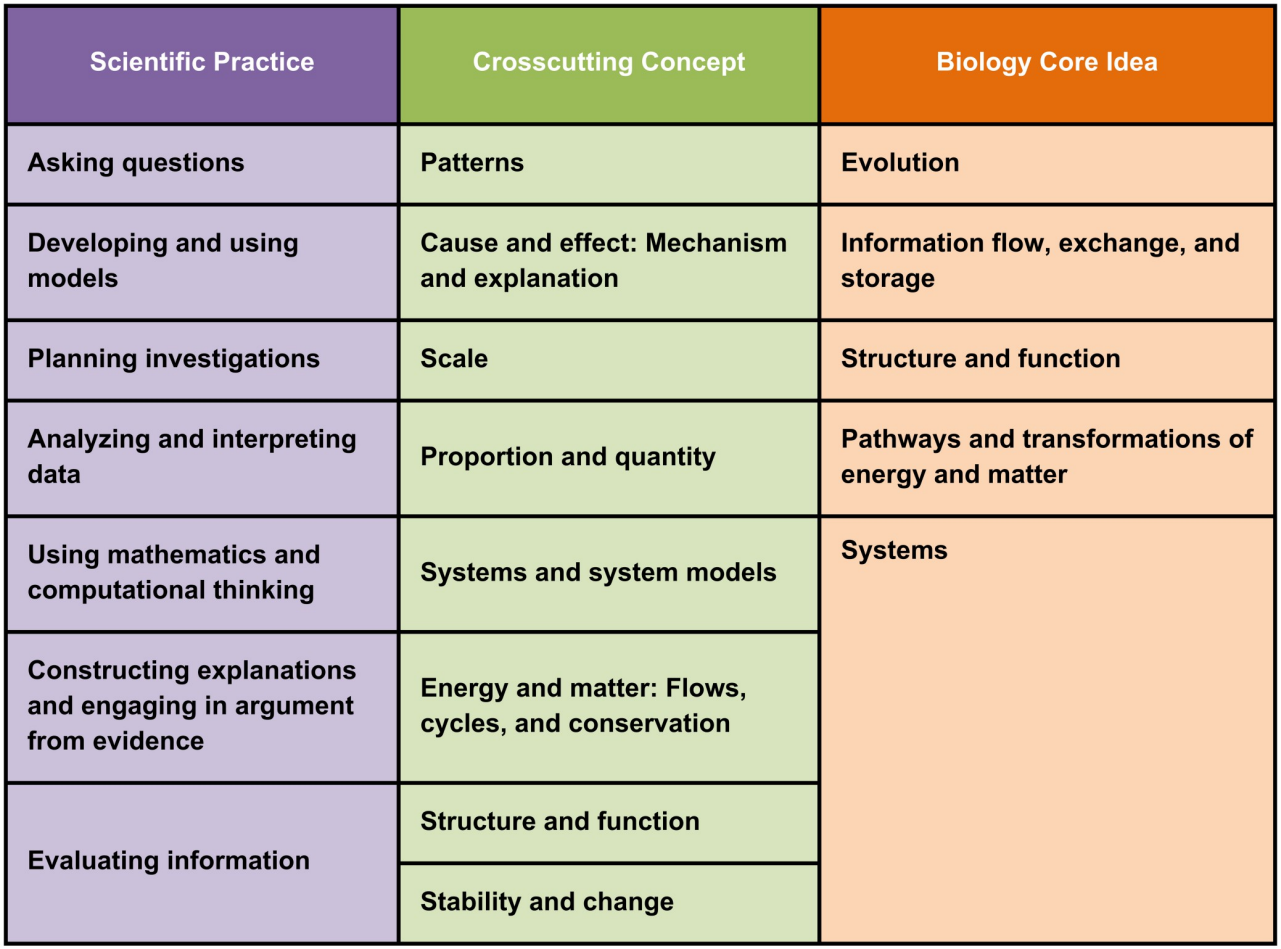
Three-dimensional framework adapted for undergraduate-level biology. Three-dimensional framework adapted from the Three-Dimensional Learning Assessment Protocol (3D-LAP) [21]. See the Methods for additional details on the adaptation of the three-dimensional framework for undergraduate biology.

Our research focuses on characterizing the extent to which undergraduate biology courses reflect the particular national call to center three-dimensional learning as the primary basis for science education [12]. The three-dimensional framework emerged from a robust synthesis of educational research [3, 9, 17–20] and builds on evidence that students develop deep understanding of science when their learning integrates three major dimensions: scientific practices (i.e., skills and processes used by scientists to establish, extend, and refine scientific knowledge), crosscutting concepts (i.e., concepts that unify the study of science through common application across disciplines), and disciplinary core ideas (i.e., a limited number of foundational concepts central to each science discipline; Fig 1). These dimensions encourage students to comprehend the nature of scientific knowledge generation via scientific practices, illuminate the unifying concepts that cut across science disciplines, and equip students with foundational core knowledge that enable subsequent acquisition of additional disciplinary content knowledge [12].

The three-dimensional framework specifies that curriculum, instruction, and assessment should enable students to actively engage in scientific practices and the application of crosscutting concepts in ways that deepen their understanding of disciplinary core ideas [12]. By focusing on deep understanding of a small number of disciplinary core ideas, three-dimensional learning may help students grasp the logic and universality of science rather than perceive science as a collection of disparate facts [12]. While the three-dimensional framework was developed for K-12 science education and is widely used in state-based science education standards [6, 22], this framework also translates to the undergraduate level and readily applies to curriculum, instruction, and assessment in the introductory-level courses that follow high school science [21, 23–26].

A recent study provided insights into faculty motivation to implement three-dimensional learning at the undergraduate level [27]. This study interviewed (n = 8) and surveyed (n = 33) faculty who participated in a two-year program aimed at helping them incorporate three-dimensional instruction into their large-enrollment science and math courses. Participants expressed strong value toward three-dimensional learning, both as an effective organizational framework and as a way to engage students in thinking like scientists. Conversely, participants reported challenges associated with understanding the framework, covering adequate content, and coordinating teaching with other instructors. While this work characterized the motivations of instructors who self-selected into a professional development program, these researchers also highlighted the need to study a broader array of instructors as well as to relate self-reported personal and contextual factors with measures of three-dimensional learning implementation.

The three-dimensional framework scaffolds science curriculum, instruction, and assessment to align with national priorities, but here we narrow our focus to exams as a tractable means to gauge three-dimensional implementation in a course. Exams are common summative assessment structures that tend to carry significant weight in undergraduate science courses [28–32]. Since what is included on exams inherently reflects what instructors intend for students to learn, exams can be used to gauge the extent to which certain content and skills have been targeted as part of the associated curriculum and instruction [10, 33, 34]. Thus, exams can provide a lens for drawing inferences about the dissemination of national calls.

**Fig 2:**
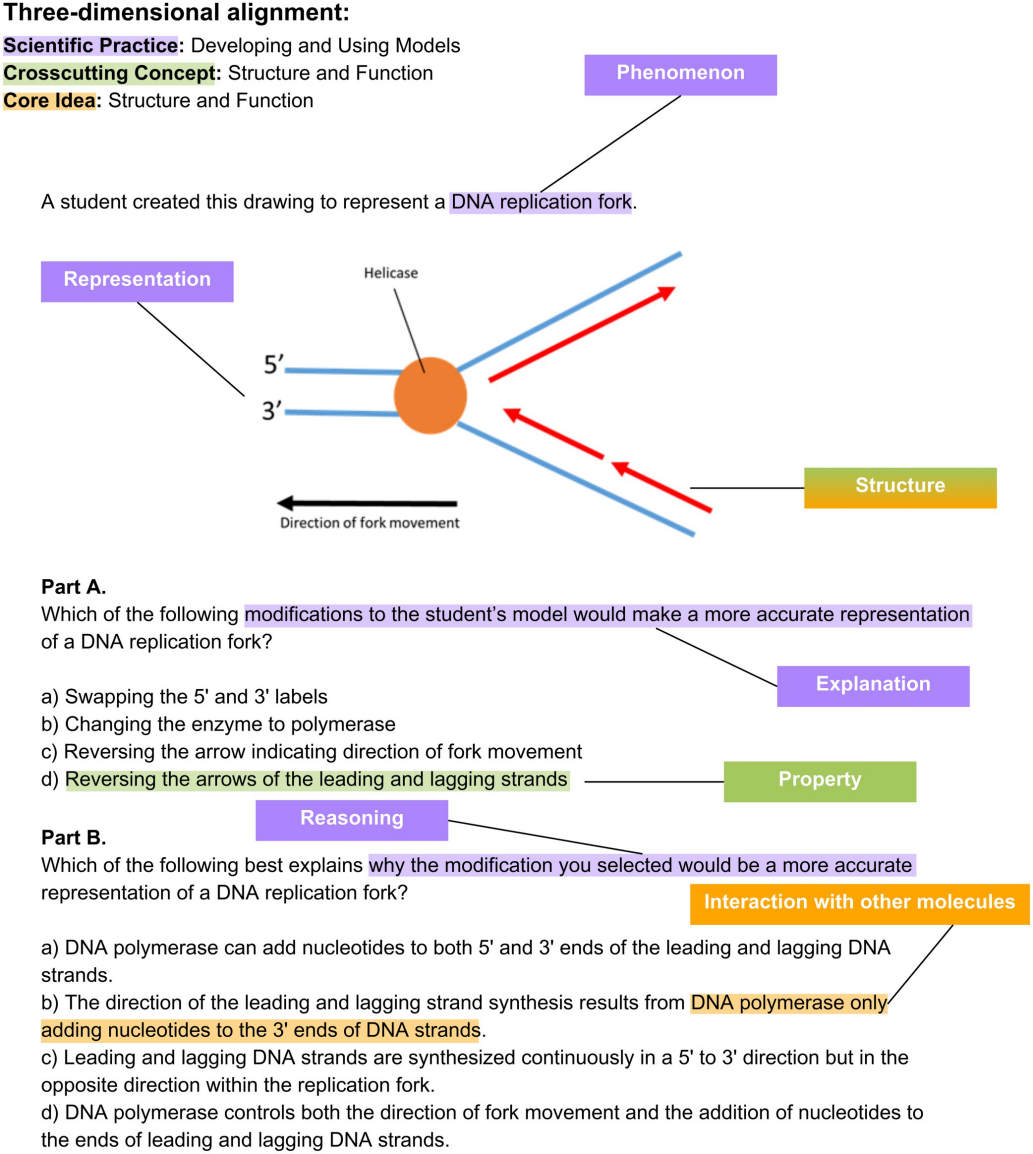
Example item coded for alignment to the three-dimensional framework. This three-dimensional example item is annotated for alignment to the 3D-LAP [21] criteria for the scientific practice “Developing and Using Models,” the 3D-LAP criteria for the crosscutting concept “Structure and Function,” and molecular-scale concepts from the BioCore Guide [39] criteria for the core concept “Structure and Function.” See S1 Table for the full description of how this item aligns to criteria for each dimension. See S2 File for additional items coded for alignment to the three-dimensional framework.

The approach of using assessments as a proxy for incorporation of the three-dimensional framework has been used in several studies [25, 35–38]. These studies applied the Three-Dimensional Learning Assessment Protocol (3D-LAP) [21] as a tool for characterizing the three-dimensional alignment of assessment items (i.e., exam questions). A common finding across these studies was that the majority of items in undergraduate science courses were not three-dimensionally aligned (see Fig 2 for an example item that is aligned to all three dimensions and S1 Table for a description of how we coded three-dimensional alignment). We previously found that only 5% of the items in our nationwide sample of lower-division (100- and 200-level) undergraduate biology exams were three-dimensional—a phenomenon largely driven by the lack of incorporation of scientific practices [35]. Scientific practices occurred in only 7% of biology exam items, as compared to crosscutting concepts and core ideas, which were present in approximately half and two-thirds of items, respectively. The low frequency of three-dimensional items raises questions about what helps or hinders implementation of the three-dimensional framework in undergraduate science [25].

### Conceptual framework for understanding how national calls relate to local practices

**Fig 3:**
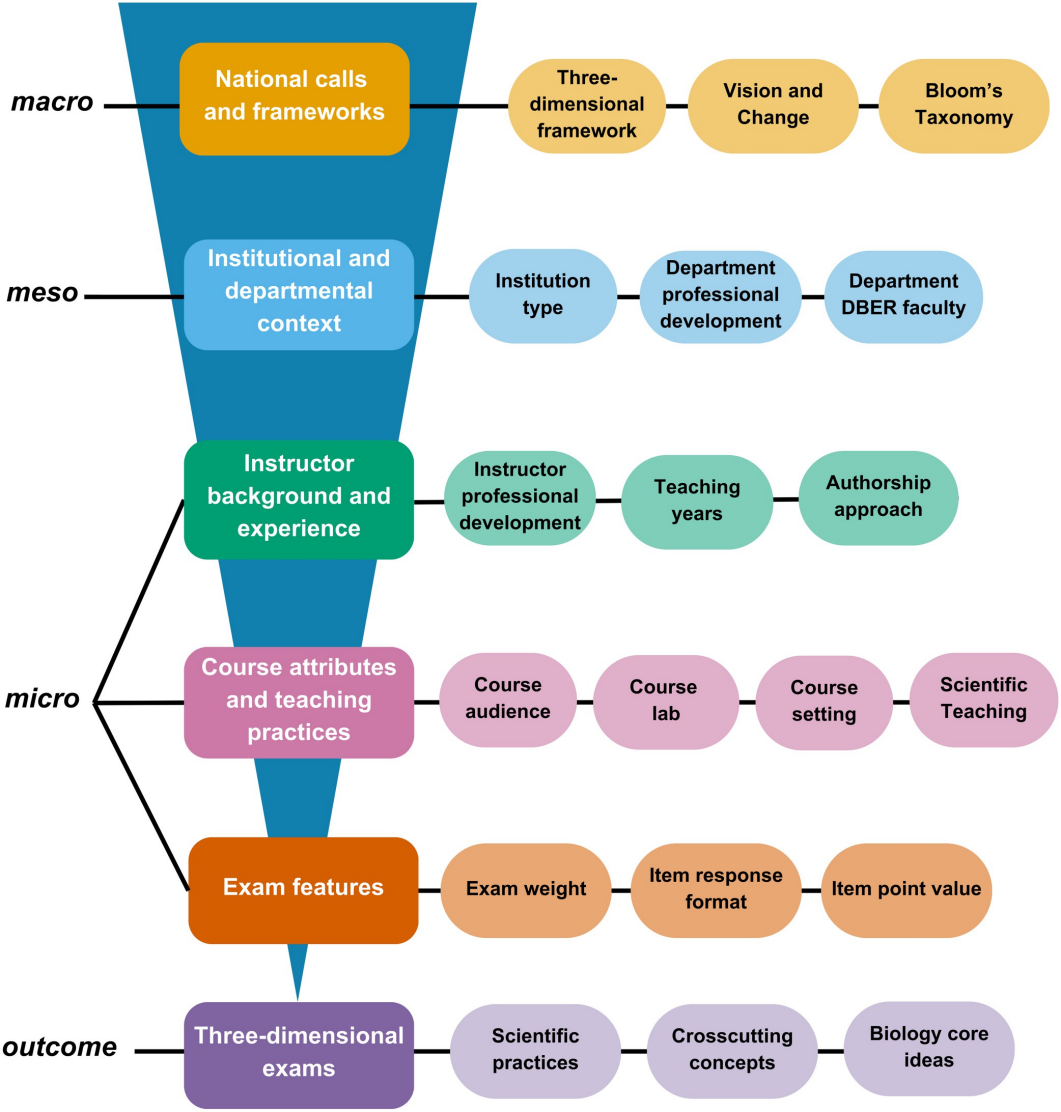
Conceptual framework of the levels of the undergraduate biology education that can influence three-dimensional assessment. We operationalize each of the levels of the education system in boxes on the left. Factors within each level are in circles to the right.

Undergraduate biology education is a complex system that spans federal agencies, professional organizations, undergraduate institutions, science departments, and biology instructors. To understand how the parts of this system interact and affect local practice in biology courses, we expand upon an existing conceptual framework that describes three contextual levels of education systems that mediate implementation of pedagogy and assessment [40, 41]. These three levels—*macro*, *meso*, and *micro—*are characterized by the span and reach of the factors associated at each level, ranging from discourse, decisions, policies, and trends at national, institutional, and instructional levels, respectively (Fig 3). Together, factors at the three levels directly or indirectly interact to influence a pedagogical outcome—in our case, three-dimensional alignment of the items on biology exams. Our conceptual framework highlights specific factors that exist at each level of the undergraduate biology education system and their potential to affect pedagogical practices particularly related to the content assessed on biology exams.

Within our conceptual framework, the *macro* level represents national-level discourse about science education that may influence assessment practices in undergraduate biology. This discourse typically occurs in the form of published documents from federal agencies and professional organizations that summarize recommendations from education researchers, issue calls to action, and present relevant educational frameworks. We highlight three particular publications for their potential to influence biology assessment practices. *A Framework for K-12 Science Education* [12] presents the three-dimensional framework that was adapted for use at the undergraduate level in the form of the Three-Dimensional Learning Assessment Protocol [21]. *Vision and Change* [4] is a national initiative aimed at reforming undergraduate biology education by emphasizing deep understanding of biology core concepts and promoting scientific reasoning through core competencies. *A Taxonomy of Educational Objectives* (i.e., Bloom’s Taxonomy) [42] is a commonly-used educational framework describing types of cognitive skills students have the potential to engage in while completing a task. By itself, Bloom’s Taxonomy is decontextualized and as such, the distinction between its cognitive skills can be unclear [43], but this framework has been adapted for use in undergraduate biology research and professional development [44–47]. Together, these three documents and their relevant adaptations represent factors that can establish norms, values, and goals for biology education.

The *meso* level aligns with institutional and departmental contexts. Factors at the *meso* level, such characteristics of the institutional environment and supports provided by the institution, are situated outside of the immediate course context but can still affect teaching practices [40, 41]. Characteristics of the institutional environment can include institution type, which reflects an institution’s function, mission, research output, and degree types [48]. Supports provided by institutions and departments may include offering professional development opportunities [49, 50], incorporating teaching assistants in high-enrollment courses [25, 51], or hiring faculty with discipline-based education research (DBER) experience in the department [52, 53]. The pedagogical decisions instructors make about their courses may be linked to whether their institutions and departments provide supports and create communities that enhance the capacity to implement instruction in line with national calls [54–57].

In our framework, the *micro* level reflects the immediate context of undergraduate biology courses. This *micro* level includes the characteristics, experiences, and practices of instructors, factors related to the student population, and the physical classroom environment [40]. Many studies in biology education focus on factors at the *micro* level, such as investigations into the effects of instructor pedagogical background [58, 59], teaching methods [60, 61], class size [62, 63], student majors [64], paired lecture and lab courses [65], and exam item format [31, 66].

### Research aims

Building on our conceptual framework, our work here uses a nationwide survey and exam collection to identify factors associated with the three-dimensional alignment of introductory undergraduate biology exams on a broad scale. We join others in recognizing the challenges associated with implementing three-dimensional assessments, in particular with eliciting explicit evidence of scientific practices [21, 27, 67–69]. Our first aim for this research is to explore potential variables associated with three-dimensional alignment from across different levels of the education system. Through this analysis, we seek to better understand what barriers may exist to three-dimensional assessment and to highlight areas in which undergraduate biology instructors may benefit from additional support. Our second aim for this research is to more fully delineate the occurrence of scientific practices in biology exams. The goal of this analysis is to achieve a higher resolution of biology exam content and to highlight where instructors may target their efforts to engage students in three-dimensional assessment.

## Methods

### Survey administration and content

The current study expands on the methods and data collection reported in our previous study [35]. Briefly, we developed an online survey through Qualtrics to collect course artifacts (e.g., a course syllabus, a summative exam, the exam answer key) along with institutional, course, and demographic information from instructors of lower-division undergraduate biology courses (i.e., 100- and 200-level courses and their equivalents). Our final dataset contained responses from 111 lower-division biology instructors at 100 unique undergraduate institutions across the United States. Our sample included broad representation from each undergraduate institution type as defined by Carnegie classifications (S2 Table) and from instructors across career stages (S3 Table). We asked instructors to complete the survey with regard to a lecture-based 100- or 200-level biology course for which they were an instructor of record. Most courses in this study were introductory-level (80%), and the remaining courses spanned a variety of lower-division biology topics including anatomy and physiology, environmental science, and microbiology (S4 Table). We relied on self-reports from survey participants that the non-introductory courses in this sample were taught as lower-division courses in their respective institutional contexts.

We also asked instructors to self-report on a series of factors potentially related to the structure and design of their exams. These factors spanned a range of levels, including connections to national calls and frameworks, institutional and departmental context, instructor background and experiences, course attributes, and exam features (Table 1). The survey items and additional descriptions are in S1 File. A descriptive summary of instructor responses to these questions is in S1 Fig. This research was classified as exempt from human-subjects review by the University of Nebraska–Lincoln (protocol 21082). Informed consent was collected electronically from participants at the start of the survey.

**Table 1:** Descriptions of factors potentially related to course exam design.

**Macro Level: Connections to national calls and frameworks**	
Use of 3D-LAP	Self-reported data about the degree to which instructors used the Three-Dimensional Learning Assessment Protocol (3D-LAP) [21] when writing their exams. Reported using a Likert scale ranging from Never to Almost Always.
Use of Vision and Change	Self-reported data about the degree to which instructors used *Vision and Change* [4] when writing their exams. Reported using a Likert scale ranging from Never to Almost Always.
Use of Bloom’s Taxonomy	Self-reported data about the degree to which instructors used Bloom’s Taxonomy [42] when writing their exams. Reported using a Likert scale ranging from Never to Almost Always.
**Meso Level: Institutional and departmental context**	
Institution type	Institutions were classified as Associate’s, Baccalaureate, Master’s, or Doctoral based on the 2018 Carnegie classifications [48].
Department professional development	Self-reported data about whether the instructor’s department has allocated resources (e.g., time or money) for faculty professional development.
Department DBER faculty	Self-reported data about whether the instructor’s department contains any faculty who identify as discipline-based education researchers (including the instructor themselves, if applicable).
**Micro Level: Instructor background and experiences**	
Instructor professional development	Self-reported data about the extent to which the instructor completed professional development about assessment (reported in 4-hour time increments).
Teaching years	Self-reported data about the number of years of teaching experience (reported in 5-year time increments).
Authorship approach	Self-reported data about whether the instructor wrote original exam items, sourced the exam items from other materials, or had a combination of both original and sourced items.
**Micro Level: Course attributes and teaching practices**	
Course audience	Self-reported data about whether the course was intended for students with STEM majors, non-STEM majors, or both STEM and non-STEM majors.
Course lab	Self-reported data about whether the course had a required lab component.
Course setting	Self-reported data about whether the course was taught in-person, online, online (because of the COVID-19 pandemic but had previously been taught in-person), or hybrid (both in-person and online).
Scientific Teaching	Self-reported data about the degree to which instructional practices aligned with Scientific Teaching principles related to active learning, data analysis and interpretation, and experimental design. Data was collected using subscales of the Measurement Instrument for Scientific Teaching (MIST) [61]. See S1 File for description of MIST score calculation.
**Micro Level: Exam features**	
Exam weight	The percentage of the final course grade that was attributed to summative exams (including midterm and final exams if applicable). Data was collected from course syllabus documents.
Item response format	Individual exam items were classified as selected-response or constructed-response based on whether students were provided a list of options to pick from or had to generate a response to the item. See S5 Table for additional details.
Item point value	The point value of individual exam items was collected from either the exam document, the associated answer key, or instructor-provided text description of their exam. Item point values were normalized across each instructor’s exam by dividing the point value of the item by the total number of points on the exam and multiplying by 100.

### Coding for item content

Our assessment dataset contained 111 exams consisting of 4337 items (i.e., test questions). We used the point values and numbering schemes specified by the instructor to determine the boundaries of individual items. In line with prior recommendations [21], we coded items that shared a common stem and/or used a sub-part numbering scheme (e.g., 2a, 2b, 2c) as a single clustered item. As exams use different grading point schemes across courses, we calculated a normalized item point value by dividing individual item point value by the total number of points on the exam and multiplying by 100. Our coding process included an initial coder training period, coding of the full dataset, and calculation of interrater reliability based on dual coding 10% of the dataset. Full details on coding procedures and interrater reliability are in our prior report [35].

We coded individual exam item content using existing protocols. Briefly, we coded scientific practices and crosscutting concepts based on the Three-Dimensional Learning Assessment Protocol (3D-LAP) [21]. We coded core ideas from the *Vision and Change* core concepts [4], as delineated in the BioCore Guide [39]. Example items meeting all three dimensions (i.e., scientific practices, crosscutting concepts, and disciplinary core ideas) are reproduced in S2 File. We coded Bloom’s Taxonomy levels [42, 70] using the Bloom’s Dichotomous Key [45]. We coded the highest Bloom’s value the item was capable of eliciting and then subsequently categorized “remember,” “understand,” and “apply” as lower-order cognitive skills (LOCS) and “analyze,” “evaluate,” and “create” as higher-order cognitive skills (HOCS). We achieved >80% agreement for coding the presence or absence of each dimension (i.e., agreement that there was or was not any scientific practice, crosscutting concept, or core idea present in the item). We had 95% agreement for coding items as LOCS versus HOCS.

### Coding for item format

We coded 13 different item formats that were classified as either constructed-response (i.e., open-ended) or selected-response (i.e., closed-ended) item types. We considered constructed-response items those that required students to generate an original response and selected-response items those that asked students to choose from a provided set of responses. Constructed-response item types included fill-in-the-blank, short answer, and essay, which were determined by the relative length of the expected student response (a single word or phrase, up to a paragraph, or multiple paragraphs, respectively). Constructed-response items also included clusters (a series of constructed-response items that shared a common stimulus or prompt), math manipulation (involving an algorithmic calculation), modeling (creating or modifying a model), and discipline-specific items (procedures, algorithms, or processes specific to biological sciences, such as manipulating genetic sequences or completing Punnett squares). Selected-response items included multiple-choice, multiple-select (a multiple-choice item in which more than one option is selected), true-false, multiple-true-false, matching, and reorder. Full descriptions of the item types coded are in S5 Table. We achieved 99% interrater agreement at the level of constructed-response versus selected-response classification, and >90% agreement for coding of each individual item format.

### Coding for partial alignment to scientific practices

The 3D-LAP coding protocol [21] provides a set of 2–4 criteria statements for each scientific practice. In the original 3D-LAP protocol, scientific practices are coded as a binary variable based on whether the item meets all the criteria statements for a given scientific practice. There is value in this binary approach to scientific practices, but we found that few instructors consistently meet the standards for full alignment. To explore underlying variation, we recoded our data on an ordinal scale based on the number of scientific practice criteria statements to which each item aligned. This scale included the categories: not aligned, partially aligned, mostly aligned, or fully aligned to a scientific practice. Briefly, items that were not aligned did not meet any of the criteria statements for a scientific practice. Items that were partially aligned met surface-level criteria, such as including a real-world biological phenomenon described in text or presented as a visual model. Items that were mostly aligned met the majority of the scientific practice criteria but lacked an explicit prompt for students to provide reasoning or justification for their thought processes. Items that were fully aligned met each criteria statement for the scientific practice. When items met criteria for multiple scientific practices, we coded the item at the highest level of alignment for each practice and conducted analysis based on the highest level across all the practices present within the item. See Fig 2 and S2 File for example items meeting each level of alignment to scientific practices. For further details on the translation of the 3D-LAP protocol into the partial alignment coding scheme, see S3 File. We achieved >80% agreement for the coding of each scientific practice (i.e., agreement about the degree of alignment to the criteria statements for each of the scientific practices).

### Statistical analysis and data availability

Following original recommendations [21], we treated three-dimensional item alignment as binary (i.e., items were either three-dimensional or not three-dimensional); thus, when three-dimensional alignment was the response variable, we used a generalized linear mixed model (GLMM) with a logit link. As we had multiple items per instructor in the sample, we included instructor as a random effect. We used forward stepwise model selection procedures based on Akaike Information Criterion (AIC) to determine the subset of variables that best explained variability in three-dimensional alignment while avoiding overfitting. Variables were individually tested for retention in the model and were only retained if the new model had an AIC value more than two units lower than the prior model [71]. We conducted statistical analysis with R statistical software [v 4.2.3] [72] using tidyverse [73] for data processing and figure generation. We used lme4 [74] for our GLMM, car [75] for calculating a type-II ANOVA for the GLMM, and multcomp [76] for Tukey post hoc comparison. De-identified data of the predictor variables retained in the GLMM and the data underlying summary statistics presented in the results are in S1 Data.

## Results

### Identifying factors associated with three-dimensional exams

Three-dimensional items engage students with a scientific practice, crosscutting concept, and core idea. We used a generalized linear mixed-effects model to identify the most salient factors associated with an item’s alignment to the three-dimensional framework. Model selection retained four predictor variables: instructor use of Bloom’s Taxonomy, institution type, item response format, and item point value (Table 2). Here, we provide additional characterization of how each of these variables relates to an instructor’s use of three-dimensional items on their exam.

**Table 2:** Analysis of deviance (Type-II Wald test) for a generalized linear mixed model with binomial logit link predicting whether an item was likely to be three-dimensionally aligned.

Fixed effects	χ^2^	*df*	*p*
Use of Bloom’s Taxonomy	5.1	1	0.02
Institution type	10.4	3	0.02
Item response format	118.8	1	< 0.001
Item point value	7.3	1	0.007

Model: Three-dimensional alignment ~ Item point value + Item response format + Institution type + Use of Bloom’s Taxonomy + (1|instructor), family = binomial(link = logit). Model *R*^*2*^ = 0.496.

See S6 Table for a Tukey post hoc comparison of institution types.

**Fig 4:**
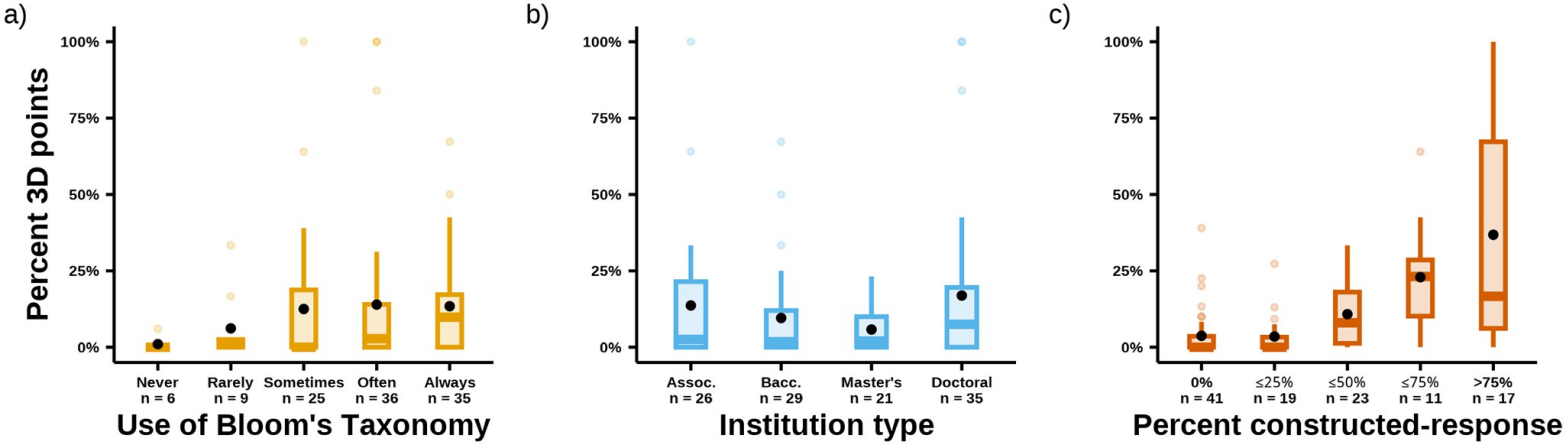
Percent of three-dimensional exam points based on instructors’ use of Bloom’s Taxonomy, institution type, and use of constructed-response items. Boxes represent the interquartile range (IQR) and whiskers represent 1.5x IQR. The solid bar represents the median value. The black dot represents the mean. a) Instructor responses to a Likert-scale survey item on reported use of Bloom’s Taxonomy when designing exams. Note that the Likert scale response “Almost Always” was abbreviated to “Always” in this figure. b) Institution types were based on Carnegie classifications. c) The point value of constructed-response items on each instructor’s exam were summed and divided by the total point value of all the exam items. The bin titles are abbreviated, and the values included in each bin are exclusive (e.g., the ≤ 75% bin includes values > 50%).

Instructors who reported higher use of Bloom’s Taxonomy when writing their exams had a higher likelihood of using three-dimensional items. While instructors who reported minimal use of Bloom’s Taxonomy had an average of 1–6% of their test points from three-dimensional items, instructors who used Bloom’s Taxonomy more frequently had 13–14% of their test points from three-dimensional items (Fig 4a). In line with this result, we further found that three-dimensional items were more likely to assess higher-order cognitive skills (χ^2^ = 1121.1, *df* = 1, p < 0.001). Approximately 68% of three-dimensional items (n = 159 of 236) assessed a higher-order cognitive skill compared to only 5% of non-three-dimensional items (n = 205 of 4101).

For institution type, we observed that the average percent of test points from three-dimensional items ranged from 6–17% (Fig 4b). Since institution type is a nominal variable, we conducted post hoc pairwise comparisons between all institution types (S6 Table). The only difference came between Doctoral and Baccalaureate institutions, with the former being slightly more associated with the use of three-dimensional items.

From the initial model, item response format stood out as the variable most closely associated with an item being three-dimensional. Exams with zero or very few constructed-response items also had a very low percent of three-dimensional points (3–4%; Fig 4c). Conversely, increased use of constructed-response items coincided with a greater percent of three-dimensional points. This culminated in exams with mostly constructed-response items (i.e., >75%, n = 17) having an average of 37% of the points from three-dimensional items.

**Fig 5:**
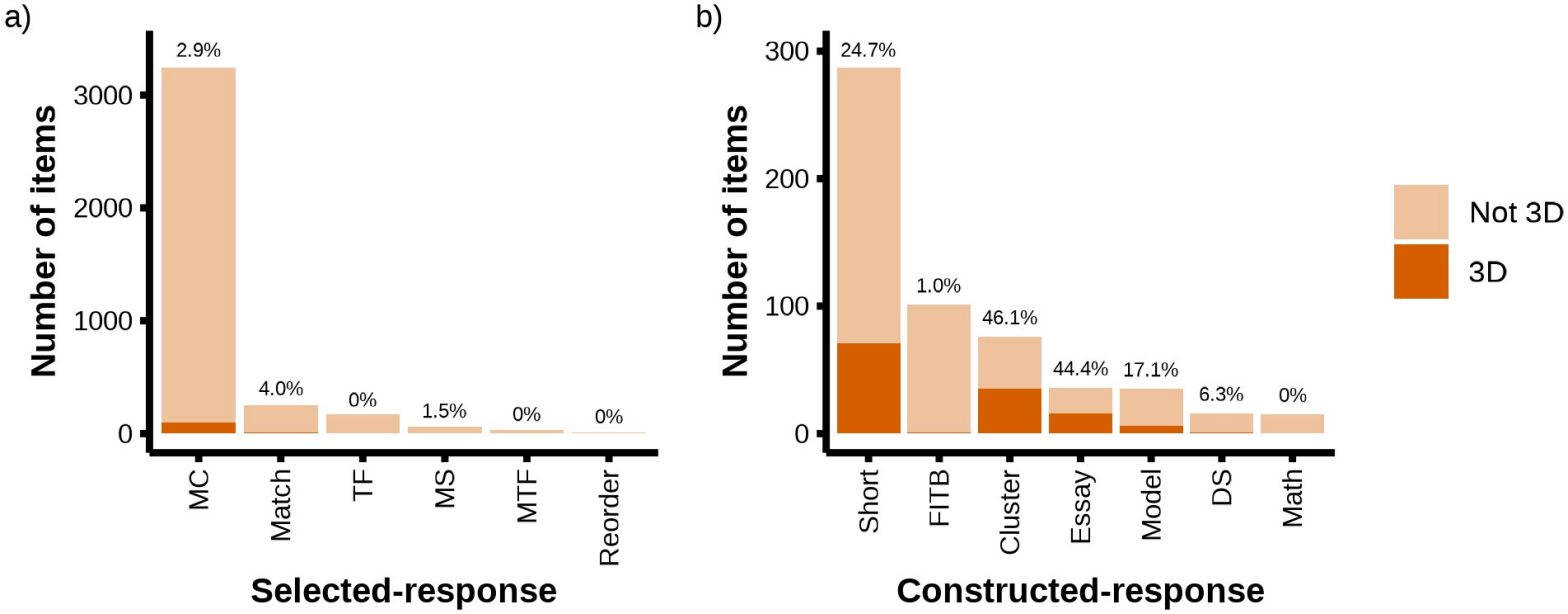
Extent to which different item types target three-dimensional learning. The percent of three-dimensional items for each item type is printed on top of each bar. a) Percent of three-dimensional selected-response items. Abbreviations: MC = multiple-choice; TF = true-false; MS = multiple-select; MTF = multiple-true-false. b) Percent of three-dimensional constructed-response items. Abbreviations: Short = short answer; FITB = fill-in-the-blank; DS = discipline-specific; Math = math manipulation.

Further analysis of the item pool helped reveal patterns in how instructors tended to use certain item formats (Fig 5, S7 Table). On the whole, selected-response items generally did not meet the three-dimensional criteria (2.8%), although it should be noted that we observed three-dimensional selected-response items (n = 106 in the item pool) comprised 45% of the total number of three-dimensional items in our sample (n = 236 in the item pool; see S2 File for examples of three-dimensional items). The low incidence of three-dimensional items was relatively consistent across specific selected-response item types (e.g., multiple-choice, matching, true-false). Conversely, constructed-response items had a much higher likelihood of being three-dimensional (23.0%). Cluster (46.1%), essay (44.4%), short answer (24.7%), and model (17.1%) formats were all amenable to being three-dimensional. While fill-in-the-blank was a common constructed-response format, only a low percent of these items targeted three-dimensions (1.0%), on par with values for selected-response formats.

Finally, three-dimensional items tended to be worth more points on exams. When normalized for the point values across an entire exam, three-dimensional items were worth 5.70 ± 0.41 SEM points on average compared to only 2.35 ± 0.03 SEM points for non-three-dimensional items.

While it is important to consider the factors that are associated with three-dimensional items, it is also important to point out variables that did not contribute to the model. Instructor use of *Vision and Change* and the 3D-LAP frameworks were both excluded. We saw no effect of department support for professional development or departments containing faculty with discipline-based education research expertise. Our model similarly excluded all factors related to instructor background and experiences (i.e., teaching years, instructor professional development, and exam authorship approach) as well as broader course attributes and teaching practices (i.e., course audience, course lab, course setting, and Scientific Teaching). Exam weight in the overall grade had no connection to the likelihood of an item being three-dimensional.

### Partial alignment to scientific practices

**Fig 6:**
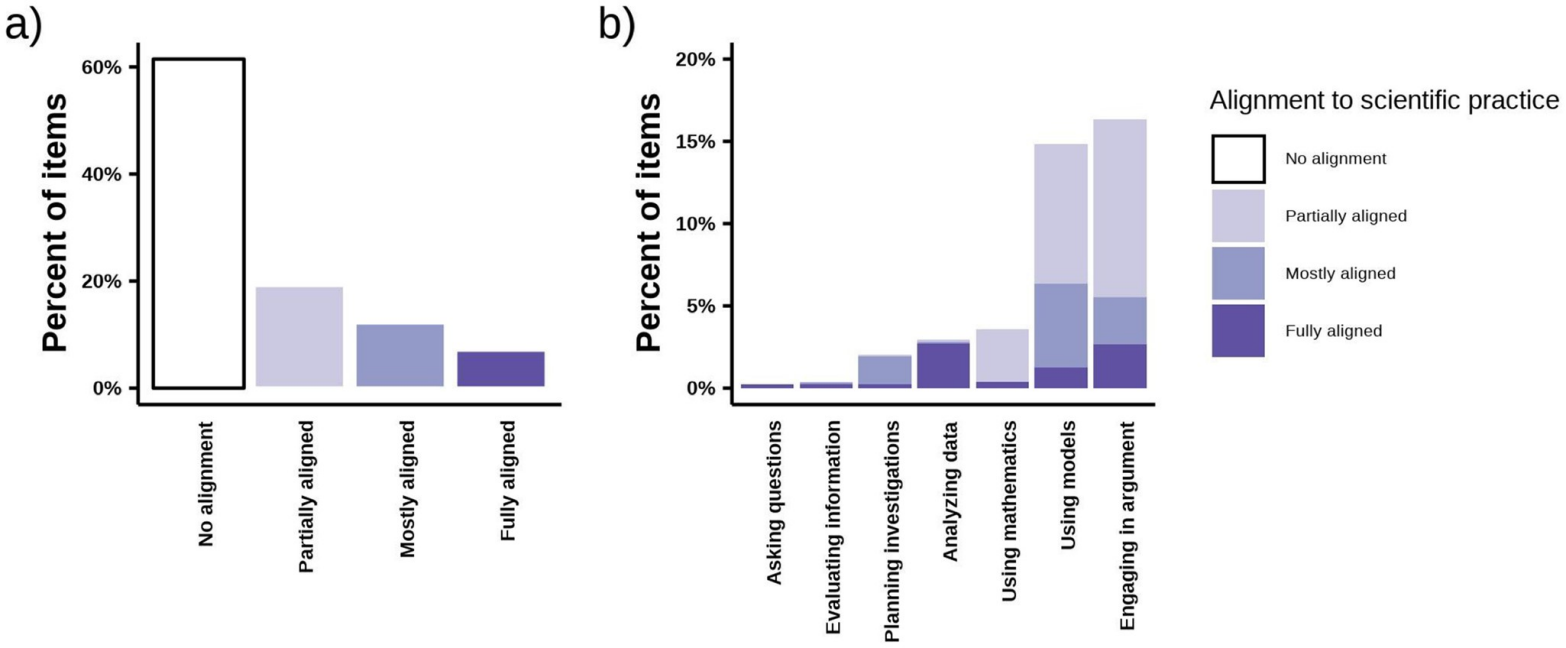
Alignment of biology exam items to 3D-LAP criteria for scientific practices. Percent of items is calculated out of the entire item pool (n = 4337). a) The highest level of alignment to any scientific practice. b) The highest level of alignment to each scientific practice, so one item may be represented in multiple columns.

We previously found that low three-dimensional occurrence was driven by the small number of items fully meeting the 3D-LAP criteria for scientific practices [35]. We subsequently hypothesized that the low incidence of scientific practices could have resulted from the 3D-LAP’s stringent criteria. To explore this hypothesis, we analyzed our data with respect to partial alignment to the scientific practice criteria statements (Fig 6). We found that even when accounting for partial alignment, most items (61%) still did not meet any of the criteria for scientific practices (Fig 6a). Approximately 19% of items were partially aligned to a scientific practice because they met surface-level criteria by including an event, observation, or observable biological phenomenon. About 12% of items were mostly aligned to a scientific practice but failed to meet full alignment because they did not ask students to explicitly convey reasoning or justification, whereas 7% of items met the fully aligned criteria.

The occurrence of partial alignment was not evenly distributed across scientific practices (Fig 6b). “Engaging in Argument,” “Using Models,” and “Using Mathematics” were the practices most likely to reach partial alignment, likely reflecting the low bar for partial alignment, which could be reached by giving students a scientific representation or claim. Meanwhile, “Using Models,” “Engaging in Argument,” and “Planning Investigations” had a relatively high incidence of items that mostly aligned but likely lacked a reasoning or justification component. When instructors had exam items that involved data analysis, they often fully met the associated scientific practice of “Analyzing and Interpreting Data.”

**Fig 7:**
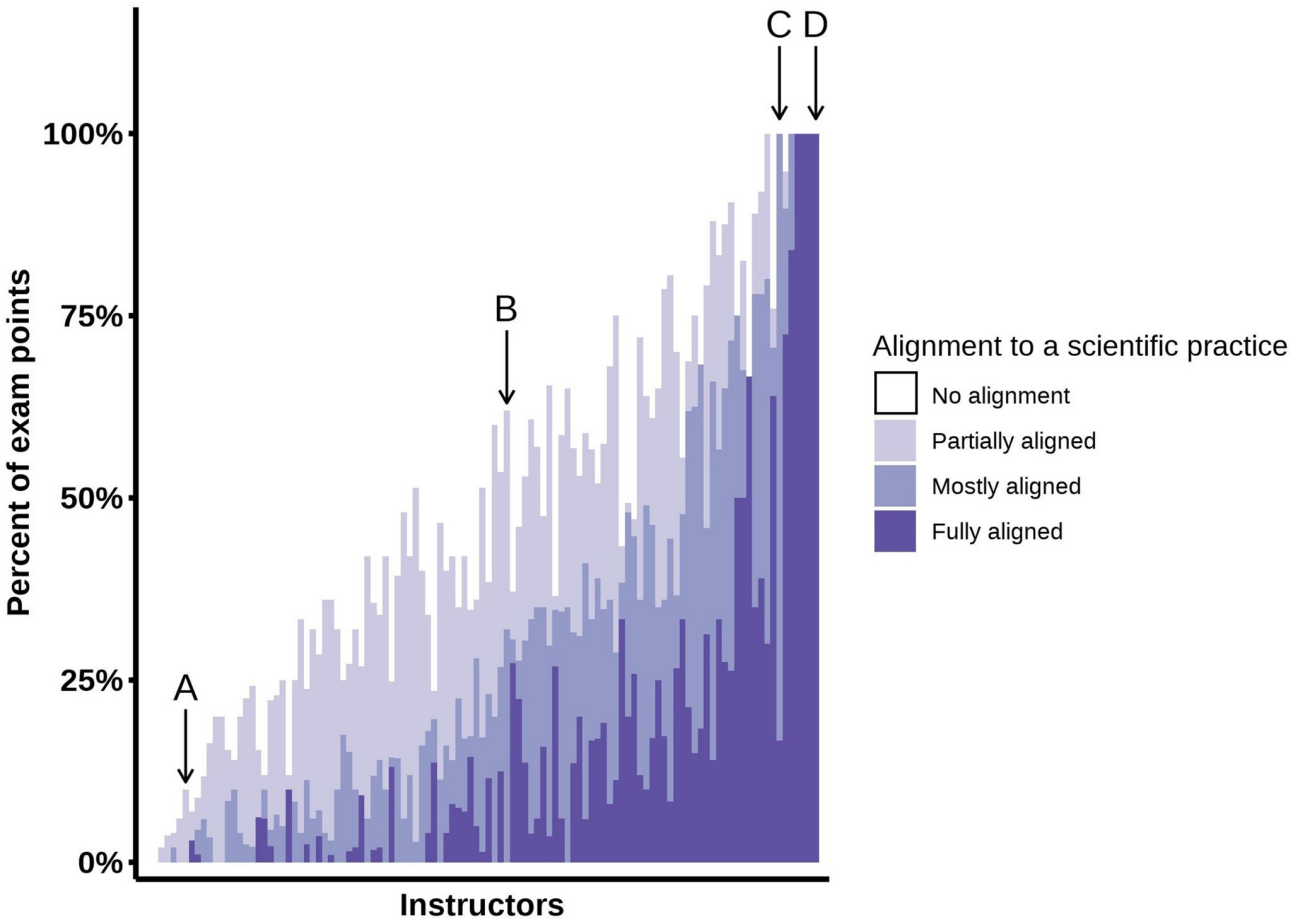
Instructor alignment of biology exams to 3D-LAP criteria for scientific practices. The point value of items at their highest level of scientific practice alignment were summed and divided by the total point value of all the exam items. To order the instructors in this graph, the highest level of scientific practice alignment for each item was recoded into a numerical scale (0 = No alignment; 0.33 = Partially aligned; 0.66 = Mostly aligned; 1 = Fully aligned) and multiplied by the normalized point value of the item and then summed for each instructor. Instructors are ordered from left to right based on increases in this summed value.

Considering exams as a whole enabled us to see the extent to which instructors incorporated aspects of scientific practices (Fig 7). While most instructors had a relatively low percent of exam points from items that fully align, many instructors also had points from items that mostly or partially meet the criteria for a scientific practice. However, there was considerable variation across instructors, with the percent of exam points partially or mostly aligned to scientific practices ranging from 0–48% and 0–83%, respectively. We further delineated this variation by describing a few representative exams (Fig 7 marked “A,” “B,” “C,” and “D”).

Exam A consisted entirely of multiple-choice items and the majority of those items tested conceptual knowledge and understanding, with an emphasis on definitions. This exam included a few items that partially aligned with the scientific practice of “Developing and Using Models,” but these items only tasked students with identifying common chemical and cellular structures from familiar models and did not ask students to extend their understanding by constructing explanations, making predictions, or engaging in reasoning about the model.

We see more novel contexts presented in Exam B, which contained non-canonical models of food webs and phylogenetic trees. Exam B did not have any items fully aligned to a scientific practice, a limitation of its entirely selected-response format that did not elicit explicit evidence of student reasoning. The greater emphasis on engaging students in novel models meant that over half of the exam points were at least partially aligned to a practice.

The importance of novel scenarios to engage students in scientific practices was again illustrated in Exam C. This exam exclusively used clusters of constructed-response items that had important elements of scientific practices, such as making calculations based on data, but these items did not connect the scientific practices back to the underlying biological phenomenon. While the knowledge and ability to perform calculations is important to biology, the scientific practice “Using Mathematics” is only fully achieved when students can interpret their calculation and demonstrate that they understand what their calculation means in the context of a scientific phenomenon.

Exam D was similarly composed of clusters of constructed-response items and was an entirely three-dimensional exam. This exam included items with models and data adapted from published scientific papers, and what distinguished this exam was its emphasis on engaging students in the process of scientific reasoning. Students were not only asked to perform calculations and interpret figures, but they were also asked to describe their logic and use their understanding of crosscutting concepts and core ideas to defend their answers. The three-dimensional items did not ask facts *about* science—they asked students to engage in the process of *doing* science.

## Discussion

Building on our conceptual framework (Fig 3), we sought to identify factors in the undergraduate biology education system associated with the incorporation of three-dimensional learning in courses, with a focus on introductory biology exams. We tested a variety of factors from across different levels and found a few factors significantly associated with three-dimensional exam alignment. Here, we propose potential explanations and implications for salient findings, and we reflect on how instructors might further achieve three-dimensional learning.

### A need for increased dissemination of the three-dimensional framework

Based on our conceptual framework, we hypothesized that *macro*-level national calls and educational frameworks have the potential to influence the content on undergraduate biology exams. We found little statistical evidence to support this hypothesis. Out of three documents—the 3D-LAP, *Vision and Change*, and Bloom’s Taxonomy—only self-reported use of Bloom’s Taxonomy was significantly associated with three-dimensional assessment. Bloom’s Taxonomy [42, 70] has been widely incorporated in undergraduate biology education research and professional development [44, 45, 47, 63, 77–83], and most instructors in our sample responded that they have used Bloom’s Taxonomy to some degree when developing their assessments (Fig 4). We suspect that instructors who intentionally targeted higher-order cognitive skills from Bloom’s Taxonomy in their assessments unintentionally met three-dimensional criteria. Instructors aiming to assess higher-order cognitive skills such as “analyze,” “evaluate,” or “create” may have also met the assessment criteria for scientific practices such as “Analyzing and Interpreting Data,” “Evaluating Information,” or “Constructing Explanations and Engaging in Argument from Evidence,” which contain the same or similar verbs. Scientific practices are rarely assessed apart from the other dimensions [35], so if an instructor met a scientific practice criteria, with or without intention, they likely also met the criteria for all three-dimensions.

Interestingly, use of *Vision and Change* and the 3D-LAP were not associated with three-dimensional assessment. A prominent movement within the biology education community, *Vision and Change* presents five core concepts and six core competencies to guide undergraduate biology courses [4]. While many instructors reported familiarity with this document (S1 Fig), the lack of connection with three-dimensional assessment may reflect a disconnect between the *macro*-level recommendations from the initial *Vision and Change* report and the *micro*-level curriculum decisions made by instructors. The 3D-LAP instrument has been used for research purposes and with smaller instructional learning communities, and our survey indicated that most biology instructors were not using this framework for assessment design.

Given these associations between educational documents and assessment practices, we speculate that targeted professional development incorporating the three-dimensional framework in course design and assessment may provide a necessary and practical entry point for instructors. Professional development on how to implement the 3D-LAP [21] and the associated Three-Dimensional Learning Observation Protocol (3D-LOP) [23] may help drive instruction and assessment to integrate scientific practices, crosscutting concepts, and disciplinary core ideas in a manner consistent with national calls. Furthermore, in contrast to Bloom’s Taxonomy, the three-dimensional framework provides additional guidance on how each dimension manifests within a science discipline, which can help instructors ensure that they are targeting and integrating the full range of practices, concepts, and core ideas.

### Three-dimensional assessment can occur at all institution types

All four institution types had instructors that found ways to incorporate three-dimensional items. This suggests that some degree of three-dimensional assessment can occur with small class sizes, such as those typical of Associate’s and Baccalaureate colleges, and agrees with previous findings that three-dimensional assessment can occur at scale in high-enrollment courses, like those commonly seen in Master’s and Doctoral universities [25, 36]. Our model retained institution type as a predictor, with a significant pairwise difference between Baccalaureate and Doctoral institutions. While this may reveal differences in underlying institutional environments, we interpret these results with caution and avoid making broad generalizations since our instructors represent only a select sample from these diverse institution classifications.

### Pedagogical resources, course attributes, and teaching practices not related to three-dimensional assessment

The final model excluded many other variables representing aspects of the education system. Among these, we found a consistent lack of connection between pedagogical resources at the *meso* and *micro* levels (e.g., departmental resources, DBER faculty, and assessment training) and three-dimensional assessment. This finding could stem from the lack of an explicit focus on the three-dimensional framework in these contexts. It also suggests that the presence of broadly-focused professional development programs may not be sufficient to initiate and sustain the implementation of national calls [84]. For example, instructors may struggle to see how three-dimensional learning fits within their existing departmental and course curricular expectations [85]. They may also be less likely to incorporate suggestions from professional development programs if they are unsure that these changes will be recognized in their departmental evaluation system [27, 83]. These findings point to the value of individual and instructional teams working together over a sustained period of time to achieve instructional change, a model that has specifically been employed to incorporate three-dimensional instruction [24, 25, 27].

A variety of *micro*-level course attributes were excluded from the model, which generally suggests that three-dimensional instruction can occur to similar degrees in a variety of courses. The equivalence of implementation across majors and non-majors courses attests to the importance of three-dimensional instruction for individuals pursuing science careers as well as those focused on non-science pathways [12]. In-person, hybrid, and online course were also similarly likely to incorporate three-dimensional assessment, suggesting that certain course formats are not disproportionately restrictive [38]. Finally, a course having a required laboratory component was not associated with the incorporation of three-dimensional assessment into the lecture exams. This finding that courses without required labs had similarly low incidence of three-dimensional assessment implies that the overall low incidence cannot be fully explained by courses partitioning scientific practices and broader three-dimensional learning into required lab courses.

We asked instructors to self-report at the *micro* level on their implementation of Scientific Teaching [82, 83, 86] using the Measurement Instrument for Scientific Teaching (MIST) [61]. We included the subcategories Active Learning Strategies, Data Analysis and Interpretation, and Experimental Design and Communication, which together closely mirror the three-dimensional framework. Our finding that MIST scores did not relate to three-dimensional assessment points to a potential misalignment between teaching and assessment practices. This misalignment may arise in more courses where science content and science practices are taught and assessed separately [87, 88]. Instructors who themselves were taught using this approach may feel unprepared to integrate three-dimensional learning across their instruction and assessment in the way envisioned by national calls [89]. Future research should consider using the Three-Dimensional Learning Observation Protocol [23], which provides a means to make a more direct comparison between observed three-dimensional teaching practices and three-dimensional assessments.

### Item format reveals constraints to three-dimensional assessment

Only a small percent (2.8%) of selected-response (i.e., closed-ended) items were three-dimensional. On one hand, these items (n = 106) provide support for the notion that multiple-choice and other selected-response formats *can* achieve the three-dimensional criteria [21, 90]. On the other hand, the infrequency of these items within the larger pool reflects that they likely require deliberate instructor development, and participants working to implement three-dimensional learning have recognized challenges with writing three-dimensional multiple-choice items [27]. It is difficult to write a selected-response item that touches on all three dimensions and elicits the reasoning needed to fulfill a scientific practice.

Constructed-response items appeared more amendable to assessing three-dimensional learning, evidenced by a higher percent (23%) of these items meeting the three-dimensional criteria. This suggests that the more flexible and generative nature of constructed-response items enables instructors to prompt students to integrate ideas across different dimensions as well as to articulate related reasoning. However, the time and resources needed to manually grade open-ended questions represents a major barrier to their implementation, particularly in large-enrollment courses.

Instructors face constraints on the amount of time they have for writing and grading questions [32], and instructors have noted that developing three-dimensional exams takes more time than standard assessments [27]. For closed-ended items, achieving three-dimensional assessment requires that instructors invest time in writing questions. Based on the items in our sample, we encourage instructors to find models and scenarios that lend themselves to asking about scientific practices and to address the highest criteria for a practice by including alternative reasoning as part of the answer options (see example question in Fig 2). Instructors may also benefit from closed-ended formats that enable multiple points of input from students, such as multiple-true-false questions [91–93] or two-tiered items (i.e., clustered items) that ask students to first answer “what” will happen in a biological scenario with a follow-up question to ask them to provide reasoning as to “how” that scenario might happen [94, 95]. For open-ended items, instructors likely need additional time or teaching assistants to help manage question grading. Efforts to use machine learning to grade open-ended items currently requires a large sample of responses beyond the scope of most courses [96, 97], but future work seems likely to make this approach increasingly plausible.

### Scientific practices as a target for three-dimensional alignment

To better understand additional avenues to three-dimensional alignment, we focused on the dimension least represented in our sample—scientific practices. Scientific practices may have been particularly low because of the 3D-LAP’s stringent coding scheme for this dimension, which requires explicit prompting for student reasoning. Such prompts encourage students to explain their logic behind scientific phenomena and provide evidence that they have appropriately engaged in a scientific practice [21, 37, 98, 99]. When an item does not explicitly ask students to provide reasoning, students may respond correctly without fully engaging in a scientific practice [37].

Our analysis of partial alignment to the scientific practices (Fig 6) revealed that most instructors had at least some components of scientific practices in their exams (Fig 7). A notable number of items were missing only the student reasoning component. This finding is not unique to biology, and previous work in chemistry has suggested that the reasoning component is often missing from assessment tasks [99, 100]. We view these items that were mostly aligned to scientific practices as promising starting places to build upon and bring into full alignment with the 3D-LAP criteria. However, not all items with some alignment to a scientific practice may be easily or directly brought into full alignment. Our sample contained many partially aligned items that only met surface-level criteria for the practices, such as including a visual representation of a biological phenomenon for students to label. These items are unlikely candidates for three-dimensional alignment. To reach three-dimensional alignment, students will need to be asked to provide evidence that they have reasoned with the depicted phenomenon and such evidence of reasoning is unlikely to be present in multiple-choice or fill-in-the-blank items in which students are only asked to identify or label parts of a familiar representation they have memorized.

### Limitations

Several limitations should be considered in the interpretation of our findings. First, we focused on exams, a common summative assessment strategy in undergraduate science courses [28–32, 101], but there are other types of summative assessments, such as projects, presentations, essays, and reports, that instructors may be using to assess scientific practices. Instructors may also be engaging students in scientific practices during formative assessments, such as in-class activities and homework assignments. Second, we focused this research on lower-division courses, which face a unique set of challenges (e.g., high enrollment, content coverage, variable student preparation) that may be barriers to the incorporation of three-dimensional instruction [32, 58, 102]. Our findings may not generalize to upper-division courses that do not feel these challenges to the same extent. Third, our survey did not specifically ask about professional development related to the three-dimensional framework, so we do not have positive evidence that targeted training will lead to implementation of the three-dimensional framework. We therefore highlight the need for the community to adopt training and support models with evidence for success [24, 25, 27] and to conduct ongoing research on how professional development can most effectively lead to implementation of three-dimensional learning and assessment.

## Conclusion

For decades, national reports [1, 4, 6, 7, 10] have called for contextualized science education that engages students in scientific practices. The three-dimensional framework [12] encapsulates many of the principles of these national calls and provides a lens for studying how national priorities are integrated across the undergraduate ology education system. Our work highlights the need for increased dissemination and adoption of the three-dimensional framework within the undergraduate biology education community. Furthermore, even with targeted training, we note that other aspects of the education system need to align with and support the implementation of the three-dimensional framework. Our research identifies the use of constructed-response items as a prominent variable with apparent ramifications. This finding speaks to ground-level challenges instructors face and reminds departments that deviation from current practice typically requires additional resources. Shifting towards three-dimensional instruction ultimately requires changes to curriculum, instruction, and assessment. For this reason, instructors may consider starting with making minor revisions to their current course materials. We view existing items as providing promising starting points for future growth, and we encourage instructors to use the 3D-LAP to increase the depth of their scientific practices and to consult other publications on adapting assessment tasks to the three-dimensional framework [21, 90].

## Supporting information

S1 Data De-identified data. (CSV)

S1 File Additional details on how factors were collected, measured, and analyzed. (DOCX)

S2 File Sample items coded for three-dimensional alignment. (DOCX)

S3 File Coding for partial alignment to scientific practices. (DOCX)

S1 Fig Distributions of responses to survey questions. (PDF)

S1 Table Alignment of example item to three-dimensional framework. (DOCX)

S2 Table Institutional Carnegie classifications and geographic regions. (DOCX)

S3 Table Self-reported demographic information of undergraduate biology instructors. (DOCX)

S4 Table Categories of lower-division biology courses included in the sample. (DOCX)

S5 Table Descriptions of item types. (DOCX)

S6 Table Tukey post hoc contrasts comparing between the probability of three-dimensional alignment based on institution type. (DOCX)

S7 Table Item types of three-dimensional and non-three-dimensional items. (DOCX)
