# Supplementary material for: Identifying factors associated with instructor implementation of three-dimensional assessment in undergraduate biology courses
Source: PLoS One. 2024 Oct 22;19(10):e0312252. doi: 10.1371/journal.pone.0312252 (PMC11495598; doi:10.1371/journal.pone.0312252)
Supplement: S1 File — (DOCX) [file pone.0312252.s002.docx]

**Identifying factors associated with instructor implementation of three-dimensional assessment in undergraduate biology courses**

Crystal Uminski, Brian A. Couch

S1 File: Additional details on how factors were collected, measured, and analyzed

This supporting information provides the survey items and additional information regarding how the survey data was processed and analyzed. Factors are listed alphabetically here, which may not reflect the order that instructors saw the items as they were presented in the original survey. Parenthetical numbers at the end of options were not seen by instructors and indicate how survey item responses were recorded. Instructor responses were retained as-is unless additional data processing is noted.

#### S1.1 Authorship approach

Survey item:

Did you write the majority of exam questions yourself?

- Yes, all by myself (1)
- Yes, by myself and with colleagues teaching the same course (2)
- No, exam questions were modified from other materials (3)
- No, exam questions were straight from other materials (4)
- Other (5) ________________________________________________

Additional data processing: Instructor responses were recoded into three categories representing original authorship (options 1 and 2) and authorship that drew from other materials (options 3 and 4) and mixed authorship indicating a combination of both original items and items from other sources. Fifteen instructors indicated option 5 (Other) and provided a text description of their authorship process, which were reviewed and recoded as “mixed authorship” or “original authorship.”

#### S1.2 Course audience

Survey item:

This course was intended for:

- STEM majors (1)
- Non-STEM majors (2)
- Both STEM majors and non-STEM majors (3)
- Other (4) ________________________________________________

Additional data processing: Instructor responses to “other” included courses intended for pre-health science students, which were recoded to “Both STEM majors and non-STEM majors.”

#### S1.3 Course lab

Survey item:

Was there a required laboratory component to this course?

- Yes (1)
- No (2)

#### S1.4 Course setting

Survey item:

At the time the exam was administered, this course was taught:

- In-person only (1)
- Online only, but previous semesters of this course were in-person (2)
- Online only and previous semesters of this course were taught online (3)
- Hybrid (i.e., contained both in-person and online components) (4)
- Other (5) ________________________________________________

Additional data processing: Six instructors selected option 5 and based on their text clarifications, these responses were re-assigned to options 1, 3, and 4.

#### S1.5 Department DBER faculty

Survey item:

Including yourself, does the department contain any faculty who identify as discipline-based education researchers (i.e., DBER faculty)?

- Yes (1)
- No (2)
- Unsure (3)

#### S1.6 Department professional development

Survey item:

Has the department allocated resources (e.g., time or money) for faculty professional development?

- Yes (1)
- No (2)
- Unsure (3)

#### S1.7 Instructor professional development

Survey item:

Approximately how many hours of professional development sessions (e.g., conference presentations, courses, workshops, or other forms of training) on the **topic of assessments** have you attended in the **past 10 years**?

- Zero hours (i.e., no professional development specific to assessments) (1)
- 1-3 hours (e.g., attending a conference presentation on assessment) (2)
- 4-8 hours (e.g., participating in a half- or full-day assessment-focused workshop) (3)
- 8-12 hours (i.e., several conference presentations, workshops, or trainings) (4)
- Greater than 12 hours (i.e., many conference presentations, workshops, or trainings) (5)

Additional data processing: The options were recoded to an ordinal scale.

Note: The bolding in this item was also in the original item presented to instructors.

#### S1.8 Scientific Teaching

Our survey contained an abbreviated version of the Measurement Instrument for Scientific Teaching (MIST; Durham et al., 2017, 2018), consisting of the items within the subcategories of Active Learning Strategies, Data Analysis and Interpretation, and Experimental Design and Communication. We applied the methods outlined in Durham et al. (2017) for converting the three MIST subcategories into a single MIST score. For each instructor, normalized responses from the MIST items were summed and divided by the number of questions, and the resulting value was multiplied by 100. The resulting MIST scores were on a 0–100 scale with higher MIST scores indicating the instructor reported using a greater amount of Scientific Teaching practices in their course.

#### S1.9 Teaching years

Survey item:

How many years of teaching experience do you have as an instructor of record?

- 0-1 year (1)
- 2-5 years (2)
- 6-10 years (3)
- 11-15 years (4)
- 16-20 years (5)
- 21-25 years (6)
- Greater than 25 years (7)

Additional data processing: The options were recoded to an ordinal scale.

#### S1.10 Use of 3D-LAP

Survey item:

To what degree do you refer to, consider, or use the following when you are constructing assessments?

|  | Never (1) | Rarely (2) | Sometimes (3) | Often (4) | Almost Always (5) |
| --- | --- | --- | --- | --- | --- |
| Three-Dimensional Learning Assessment Protocol (3D-LAP) |  |  |  |  |  |

Additional data processing: The options were recoded to an ordinal scale.

#### S1.11 Use of Bloom’s Taxonomy

Survey item:

To what degree do you refer to, consider, or use the following when you are constructing assessments?

|  | Never (1) | Rarely (2) | Sometimes (3) | Often (4) | Almost Always (5) |
| --- | --- | --- | --- | --- | --- |
| Bloom’s Taxonomy |  |  |  |  |  |

Additional data processing: The options were recoded to an ordinal scale.

#### S1.12 Use of Vision and Change

Survey item:

To what degree do you refer to, consider, or use the following when you are constructing assessments?

|  | Never (1) | Rarely (2) | Sometimes (3) | Often (4) | Almost Always (5) |
| --- | --- | --- | --- | --- | --- |
| Recommendations made by the *Vision and Change* report |  |  |  |  |  |

Additional data processing: The options were recoded to an ordinal scale.
