# Supplementary material for: Identifying factors associated with instructor implementation of three-dimensional assessment in undergraduate biology courses
Source: PLoS One. 2024 Oct 22;19(10):e0312252. doi: 10.1371/journal.pone.0312252 (PMC11495598; doi:10.1371/journal.pone.0312252)
Supplement: S2 File — (DOCX) [file pone.0312252.s003.docx]

**Identifying factors associated with instructor implementation of three-dimensional assessment in undergraduate biology courses**

Crystal Uminski, Brian A. Couch

S2 File. Sample items coded for alignment to the three-dimensional framework

Sample Item #1

Scientific Practice: Analyzing and Interpreting Data
Crosscutting Concept: Cause and Effect
Core Idea: Evolution
Bloom’s Taxonomy Level: Evaluate

Cause

The invasive cane toad was introduced to Australia in the 1930s. The toads are extremely poisonous to red-bellied black snakes, and there was some concern that the snake population might be decimated. However, the snakes and toads seem to be co-existing. Imagine a biologist began investigating the situation by surveying red-bellied black snakes in areas with and without cane toads and found one major difference between the two populations. The results are depicted in the hypothetical graph below.


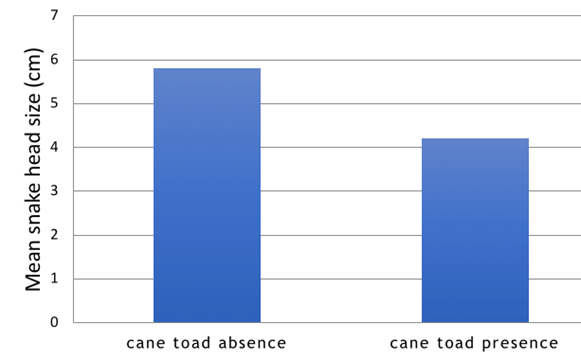


3. Interpretation

Effect

Evaluate

2. Data

1. Claim

Which of the following scenarios is most likely given the data?

1. Where cane toads are present, red-bellied black snakes evolved a tolerance to cane
   toad toxins that allowed them to survive and reproduce.

Mechanism

1. Where cane toads are present, red-bellied black snakes with large heads died while
    those with small heads survived and reproduced.
2. The presence of cane toads caused mutations that resulted in smaller heads.

Evolution

1. The red-bellied black snakes mutated in response to the cane toads so that they could
   tolerate cane toad toxins.

| **Applied coding protocol for Sample Item #1** | | |
| --- | --- | --- |
| **Code** | **Criteria** | **Rationale** |
| Scientific Practice: Analyzing and Interpreting Data | 1. Question gives a scientific question, claim, or a hypothesis to be investigated.  2. Question gives a representation of data (table, graph, list of observations, etc.) provided to answer the question or test the claim or hypothesis.  3. Question asks student to select an interpretation of the results or an assessment of the validity of the conclusions in the context of the scientific question, claim, or hypothesis. | 1. Question gives a scientific claim about differences in populations of red-bellied black snakes in the presence of cane toads.  2. Question gives a representation of data about the mean head size of red-bellied black snakes in the presence and absence of cane toads.  3. Question asks student to select an appropriate interpretation and draw conclusions about the red-bellied black snake head size data. |
| Crosscutting Concept: Cause and Effect | The question provides at most two of the following: 1) a cause, 2) an effect, and 3) the mechanism that links the cause and effect, and the student is asked to provide the other(s). | Cause: Presence of cane toads Effect: Change in mean snake head size Mechanism: Differential survival of snakes based on head size |
| Core Idea: Evolution | The characteristics of populations change over time due to changes in allele frequencies. Changes in allele frequencies are caused by random and nonrandom processes – specifically mutation, natural selection, gene flow, and genetic drift. Not all of these changes are adaptive. | The characteristics of the red-bellied black snake populations change over time because of non-random processes – specifically natural selection. |
| Bloom’s Taxonomy Level: Evaluate | Students have to interpret data (graph, table, figure, story, problem, etc.) then determine whether the data are consistent with a given scenario or whether conclusions are consistent with the data, critique validity, quality, or experimental data/methods, or make a judgment and/or justify their answer. | Students have to interpret data about mean snake head size then determine whether the data are consistent with a given scenario. |

Sample three-dimensional item with alignment to the scientific practice, crosscutting concept, core idea, and Bloom’s Taxonomy level indicated within the item and justified in a table.


**Sample Item #2**

Scientific Practice: Constructing Explanations and Engaging in Argument from Evidence
Crosscutting Concept: Cause and Effect
Core Idea: Evolution, Information Flow
Bloom’s Taxonomy Level: Analyze

1. Phenomenon

Recently, two chiropractors in Queensland, Australia published a research article stating that the external occipital protuberance (AKA: EOP or horn-like bone spurs) are growing on human skulls due to “sustained aberrant postures associated with the emergence and extensive use of hand-held contemporary technologies, such as smartphones and tablets.”^1,2^

Effect

Cause

To put it simply, the “horns” are said to be growing at the bottom of the skull, where the head bends to look down at a cell phone, and the authors say that human bodies are physically adapting to use modern technology.

Is this an example of natural selection or evolution, and why?

Information flow

2, 3. Make a claim based on scientific principles

4. Reasoning

Evolution

Mechanism

Analyze

Notes:

^1^Shahar, D. & Sayers, M. G. L. (2018). Prominent exostosis projecting from the occipital squama more substantial and prevalent in young adult than older age groups. *Scientific Reports*, 8, ar:3354.

^2^See also: Shahar, D. & Sayers, M. G. L. (2019). Author Correction: Prominent exostosis projecting from the occipital squama more substantial and prevalent in young adult than older age groups. *Scientific Reports, 9*, ar:13707.

| **Applied coding protocol for Sample Item #2** | | |
| --- | --- | --- |
| **Code** | **Criteria** | **Rationale** |
| Scientific Practice: Constructing Explanations and Engaging in Argument from Evidence | 1. Question gives an event, observation, or phenomenon.  2. Question gives or asks student to make a claim based on the given event, observation, or phenomenon.  3. Question asks student to provide scientific principles or evidence in the form of data or observations to support the claim.  4. Question asks student to provide reasoning about why the scientific principles or evidence support the claim. | 1. Question gives the phenomenon of the external occipital protuberance (EOP).  2. Question asks student to make a claim about the EOP.  3. Question asks students to justify claim about EOP using scientific principles of natural selection and evolution.  4. Question asks students to provide reasoning to support their claim about the EOP. |
| Crosscutting Concept: Cause and Effect | The question provides at most two of the following: 1) a cause, 2) an effect, and 3) the mechanism that links the cause and effect, and the student is asked to provide the other(s). | Cause: Extensive use of smartphones and tablets  Effect: Growth of EOP  Mechanism: Non-heritable change in phenotype |
| Core Idea: Evolution | Species evolve over time, and new species can arise, when allele frequencies change due to mutation, natural selection, gene flow, and genetic drift. | Students distinguish that the EOP is not an example of natural selection or evolution. |
| Core Idea: Information Flow | A genotype influences the range of possible phenotypes in an individual; the actual phenotype results from interactions between alleles and the environment. | The EOP phenotype is the result of interactions with the environment (smartphone use). |
| Bloom’s Taxonomy Level: Analyze | Students are asked to compare/contrast information, have to interpret data (graph, table, figure, story problem, etc.) and come to a conclusion about the data mean (they may or may not be required to explain the conclusion), and/or have to decide what data are important to solve the problem (i.e., picking out relevant from irrelevant information). | Students compare the information about EOP to their understanding of natural selection and evolution and explain their conclusion. |

Sample three-dimensional item with alignment to the scientific practice, crosscutting concept, core idea, and Bloom’s Taxonomy level indicated within the item and justified in a table.

**Sample Item #3**

Scientific Practice: Developing and Using Models
Crosscutting Concept: Energy and Matter: Flows, Cycles, and Conservation
Core Idea: Transformations of Energy and Matter
Bloom’s Taxonomy Level: Evaluate

Energy and matter

1. Phenomenon

Minerals in the soil move into a plant root cell via facilitated diffusion. Image 4 is meant to show this process of a mineral shown in red moving in the direction of the black arrow across the membrane in the green through a membrane protein in blue. However, something is wrong with the image. What is wrong with the image? (select all that apply)

3. Explanation


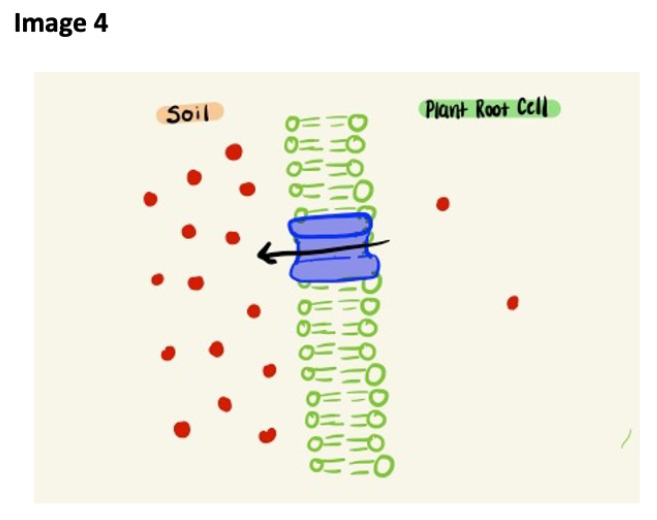


2. Representation

Evaluate

Energy and matter

4. Reasoning

Transfer of matter

▢ The minerals are moving in the wrong direction, they should be moving down their concentration gradient (from an area of high concentration to low concentration)

▢ Minerals should not be shown being taking in by the roots of the plant, minerals only enter the plant via the stomata

▢ ATP should be shown in the image along the membrane protein to show that ATP is used for facilitated diffusion

| **Applied coding protocol for Sample Item #3** | | |
| --- | --- | --- |
| **Code** | **Criteria** | **Rationale** |
| Scientific Practice: Developing and Using Models | 1. Question gives an event, observation, or phenomenon for the student to explain or make a prediction about.  2. Question gives a representation or asks student to select a representation.  3. Question asks student to select an explanation for or prediction about the event, observation, or phenomenon.  4. Question asks student to select the reasoning that links the representation to their explanation or prediction. | 1. Question gives phenomenon of soil minerals moving into plant root cells.  2. Question provides Image 4 as a representation of a plant root cell.  3. Question asks student to select an explanation for how Image 4 is an incorrect representation of the phenomenon.  4. Question asks student to select an answer with appropriate reasoning. |
| Crosscutting Concept: Energy and Matter: Flows, Cycles, and Conservation | To code an assessment task with Energy and Matter: Flows, Cycles, and Conservation, the question asks the student to describe the transfer or transformation of energy or matter within or across systems, or between a system and its surroundings. | Question asks student to describe the transfer of minerals between a system (a plant root cell) and its surroundings (soil). |
| Core Idea: Transformations of Energy and Matter | Intracellular and intercellular movement of molecules occurs via 1) energy-demanding transport processes and 2) random motion. A molecule’s movement is affected by its thermal energy, size, electrochemical gradient, and biochemical properties. | Question asks students to consider molecular movement in relation to the concentration gradient. |
| Bloom’s Taxonomy Level: Evaluate | Students have to interpret data (graph, table, figure, story, problem, etc.) then determine whether the data are consistent with a given scenario or whether conclusions are consistent with the data, critique validity, quality, or experimental data/methods, or make a judgment and/or justify their answer. | Students interpret data about the concentration gradient presented in Image 4 and determine whether the data are consistent with the given situation (facilitated diffusion). |

Sample three-dimensional item with alignment to the scientific practice, crosscutting concept, core idea, and Bloom’s Taxonomy level indicated within the item and justified in a table.

**Sample Item #4**

Scientific Practice: Analyzing and Interpreting Data
Crosscutting Concept: Patterns
Core Idea: Information Flow
Bloom’s Taxonomy Level: Evaluate

3. Interpretation

1. Claim

This figure shows data from a study of the heritability of weight and fin length in fish, finding a heritability of 0.2 for weight and a heritability of 0.8 for fin length. Which of the following statements is NOT supported by the figures and those findings?

2. Data


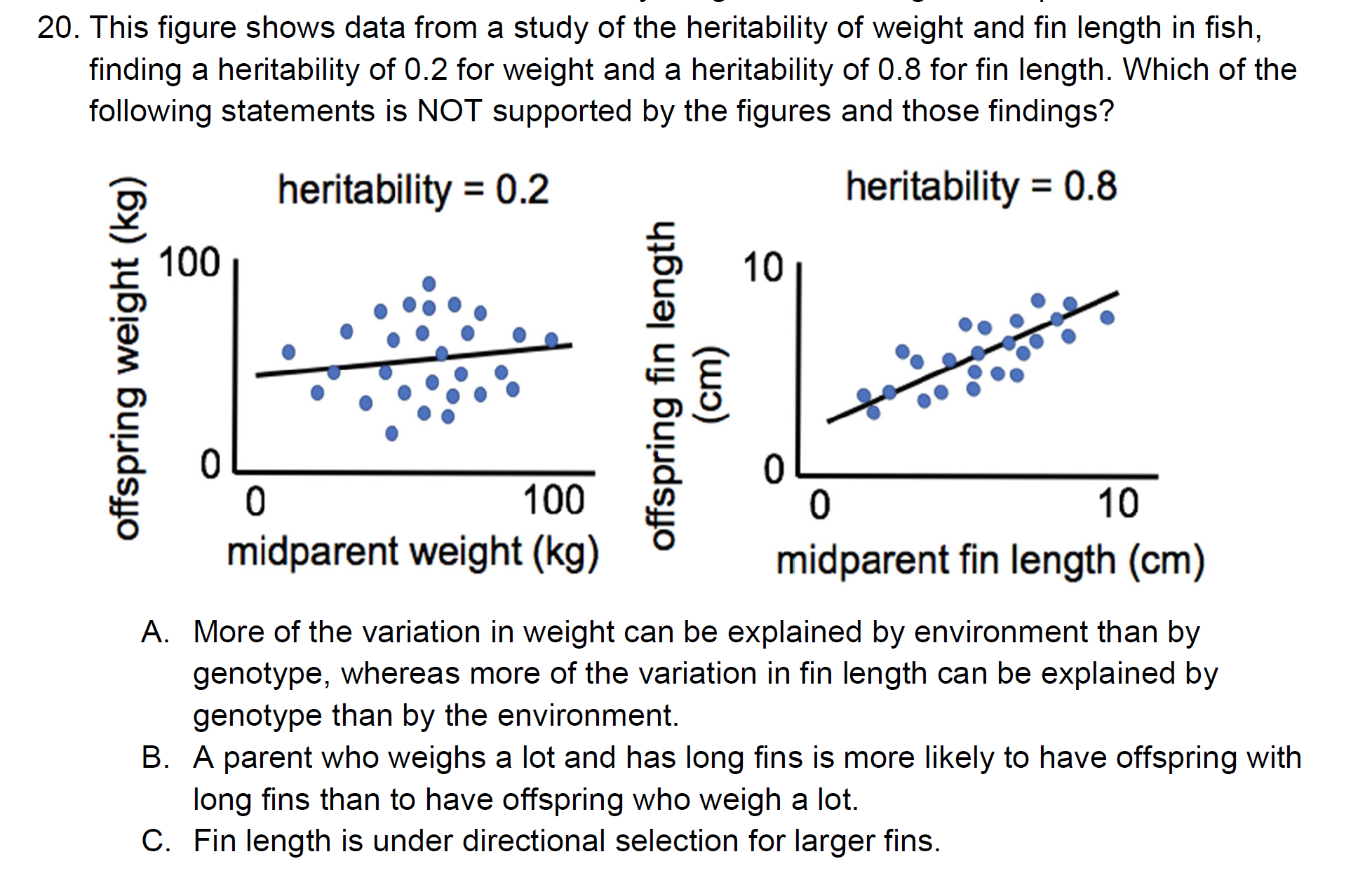


Pattern

Evaluate

1. More of the variation in weight can be explained by environment than by genotype, whereas more of the variation in fin length can be explained by genotype than by the environment.
2. A parent who weighs a lot and has long fins is more likely to have offspring with long fins than to have offspring who weighs a lot.
3. Fin length is under directional selection for larger fins.

Information flow

| **Applied coding protocol for Sample Item #4** | | |
| --- | --- | --- |
| **Code** | **Criteria** | **Rationale** |
| Scientific Practice: Analyzing and Interpreting Data | 1. Question gives a scientific question, claim, or a hypothesis to be investigated.  2. Question gives a representation of data (table, graph, list of observations, etc.) provided to answer the question or test the claim or hypothesis.  3. Question asks student to select an interpretation of the results or an assessment of the validity of the conclusions in the context of the scientific question, claim, or hypothesis. | 1. Question gives scientific claim about the heritability of weight and fin length in fish.  2. Question provides graphs showing the relationship between parent and offspring weight and fin length.  3. Question asks student to interpret which conclusions about heritability are not supported by the data. |
| Crosscutting Concept: Patterns | To code an assessment task with Patterns, the question asks the student to identify patterns or trends emerging from three or more events, observations, or data. | Question asks student to use patterns in the graphs to make claims about heritability of weight and fin length. |
| Core Idea: Transformations of Energy and Matter | Individuals transmit genetic information to their offspring; A genotype influences the range of possible phenotypes in an individual; the actual phenotype results from interactions between alleles and the environment. | Question asks student to consider the relationship between parent and offspring phenotypes for weight and fin length and how variation in these traits may be explained by the environment. |
| Bloom’s Taxonomy Level: Evaluate | Students have to interpret data (graph, table, figure, story, problem, etc.) then determine whether the data are consistent with a given scenario or whether conclusions are consistent with the data, critique validity, quality, or experimental data/methods, or make a judgment and/or justify their answer. | Students interpret data about heritability presented in the graphs and determine which option is not consistent with the data. |

Sample three-dimensional item with alignment to the scientific practice, crosscutting concept, core idea, and Bloom’s Taxonomy level indicated within the item and justified in a table.

**Sample Item #5**

Scientific Practice: Asking Questions
Crosscutting Concept: Proportion and Quantity
Core Idea: Evolution
Bloom’s Taxonomy Level: Create

In certain species of birds, the males with longer tail feathers tend to have a greater number of active nests, but these males with long tails face additional costs associated with movement and resource acquisition and may experience higher rates of predation. Based on this observation, researchers conducted a tail manipulation experiment in a species of bird that do not naturally produce long tails. The researchers first measured the natural tail length of males in the population (Figure 1). Then researchers randomly selected some male birds and elongated the tail feathers by cutting and gluing the long tail feathers from another similar species of bird with naturally long tails. To control for the effects of tail manipulation, all male birds had their tail feathers cut and glued, even if their tail was not elongated. After the tail manipulation, the researchers measured the number of active nests each male had in its territory (Figure 2)^1^.

1. Data

Proportion and Quantity

Based on this passage:

2. Testable question

1. Identify the hypothesis of the researchers’ study.
2. Describe the results in Figures 1 and 2.
3. Explain whether the results in Figures 1 and 2 support or refute the hypothesis.

Evolution

1. Identify the type of selection occurring in this population.
2. Generate a new research question that could be tested with a similar tail manipulation experimental design and use your understanding of selection to hypothesize what results you might expect to observe.

Create

Notes:

^1^Data based on Pryke, S. R., & Andersson, S. (2002). *Proc. R. Soc. Lond. B.* 269, 2141-2146.

| **Applied coding protocol for Sample Item #5** | | |
| --- | --- | --- |
| **Code** | **Criteria** | **Rationale** |
| Scientific Practice: Asking Questions | 1. Question gives an event, observation, phenomenon, data, scenario, or model.  2. Question asks student to generate an empirically testable question about the given event, observation, phenomenon, data, scenario, or model. | 1. Question gives the phenomenon and associated data for the relationship between tail feather length and the number of active nesting sites.  2. Question asks student to generate a testable research question about tail length in birds. |
| Crosscutting Concept: Proportion and Quantity | To code an assessment task with Proportion and Quantity, the question asks the student to predict the response of one variable to changes in another or identify the relationship between two or more variables from data. | Question asks student to use data to determine the relationship between the variables of tail length and number of active nest sites. |
| Core Idea: Evolution | Fitness is an individual’s ability to survive and reproduce. It is environment-specific and depends on both abiotic and biotic factors. Evolution of optimal fitness is constrained by existing variation, trade-offs and other factors. | Question asks student to consider fitness of male birds based the trait of tail length. |
| Bloom’s Taxonomy Level: Create | To code for Create/Synthesize, students must be synthesizing information into a bigger picture (coherent whole) or creating something they haven’t seen before (a novel hypothesis, a novel model, etc.), building up a model or novel hypothesis from data, or putting information from several areas together to create a new pattern/structure/model/etc. | Question asks student to generate a novel, testable research question about tail length in birds. |

Sample three-dimensional item with alignment to the scientific practice, crosscutting concept, core idea, and Bloom’s Taxonomy level indicated within the item and justified in a table.
