# Supplementary material for: Identifying factors associated with instructor implementation of three-dimensional assessment in undergraduate biology courses
Source: PLoS One. 2024 Oct 22;19(10):e0312252. doi: 10.1371/journal.pone.0312252 (PMC11495598; doi:10.1371/journal.pone.0312252)
Supplement: S3 File — (DOCX) [file pone.0312252.s004.docx]

**Identifying factors associated with instructor implementation of three-dimensional assessment in undergraduate biology courses**

Crystal Uminski, Brian A. Couch

S3 File: Coding for partial alignment to scientific practices

Our coding for partial alignment to the 3D-LAP scientific practices criteria accounts for the inconsistent number of criteria statements between the different scientific practices. When the scientific practice had three criteria statements (e.g., “Planning Investigations,” “Using Mathematics and Computational Thinking,” or “Evaluating Information”), we coded alignment to each statement, where meeting zero, one, two, or three statements was coded as no alignment, partially aligned, mostly aligned, and fully aligned to the scientific practice, respectively.

The 3D-LAP criteria for the scientific practice “Asking Questions” only contained two criteria statements, so we coded alignment as either no alignment or fully aligned. The first criterion (“Question gives an event, observation, phenomenon, data, scenario, or model”) was similar to the first criterion of multiple practices and could not accurately be coded at the level of that statement.

In cases where the 3D-LAP contained four criteria statements for the scientific practice (e.g., “Developing and Using Models,” “Analyzing and Interpreting Data,” “Constructing Explanations and Engaging in Argument from Evidence”), we did not code at the level of the first criterion. In each case when there were four criteria statements, the first criterion could be met by providing an event, observation, phenomenon, or hypothesis, and as such could not be distinguished between scientific practices that shared the same or similar criteria. When there were four criteria for the scientific practice, we coded alignment to zero, two, three, or four statements as no alignment, partially aligned, mostly aligned, and fully aligned to a scientific practice, respectively.
