# Supplementary material for: Identifying factors associated with instructor implementation of three-dimensional assessment in undergraduate biology courses
Source: PLoS One. 2024 Oct 22;19(10):e0312252. doi: 10.1371/journal.pone.0312252 (PMC11495598; doi:10.1371/journal.pone.0312252)

National calls and frameworks

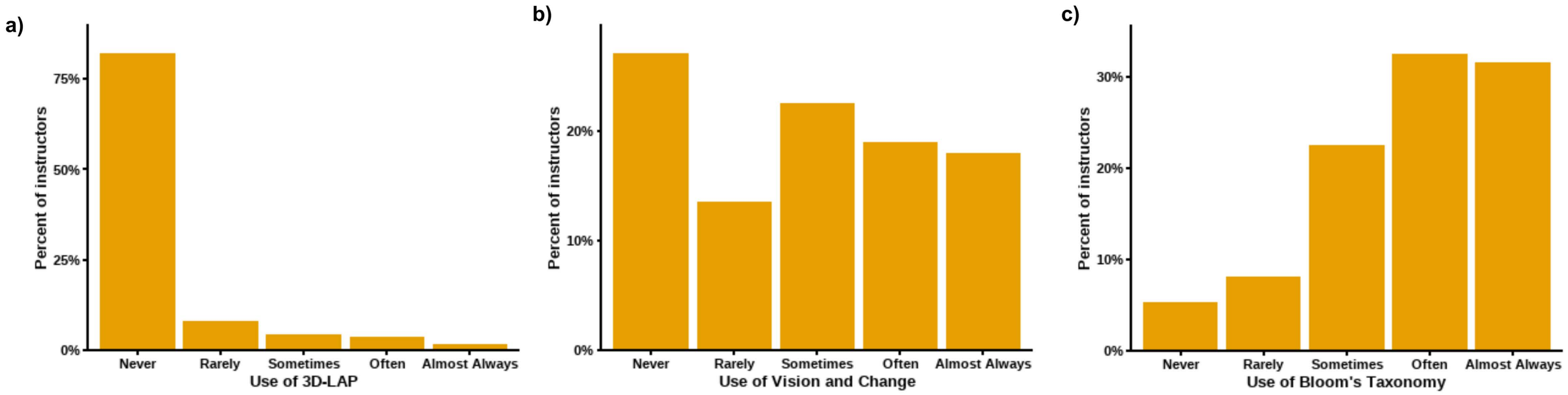

Institutional and departmental context

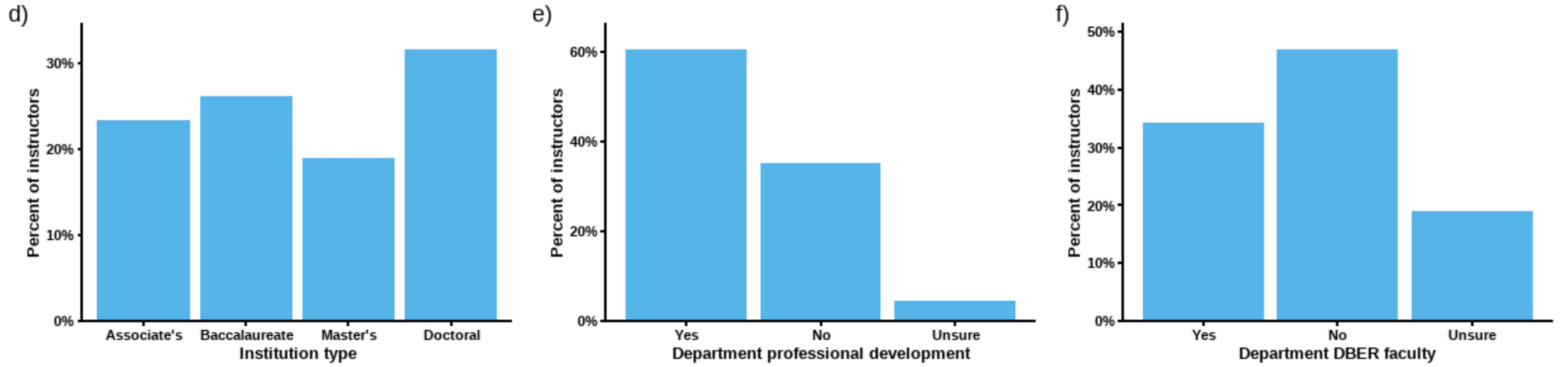

Instructor background and experience

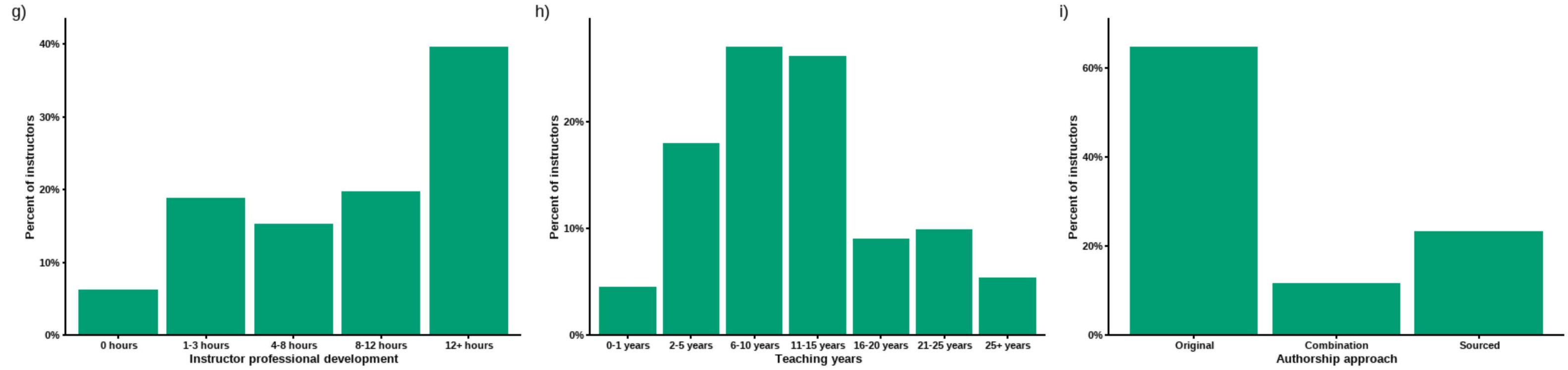

Course attributes and teaching practices

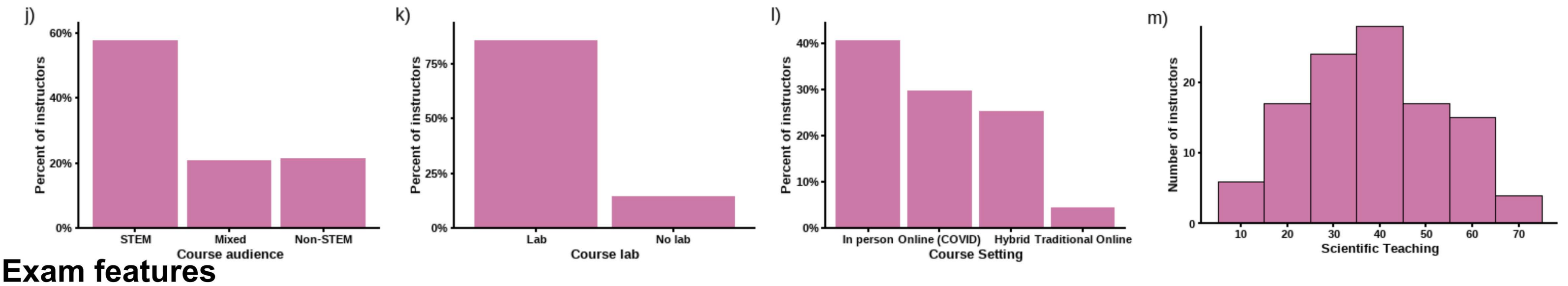

Exam features

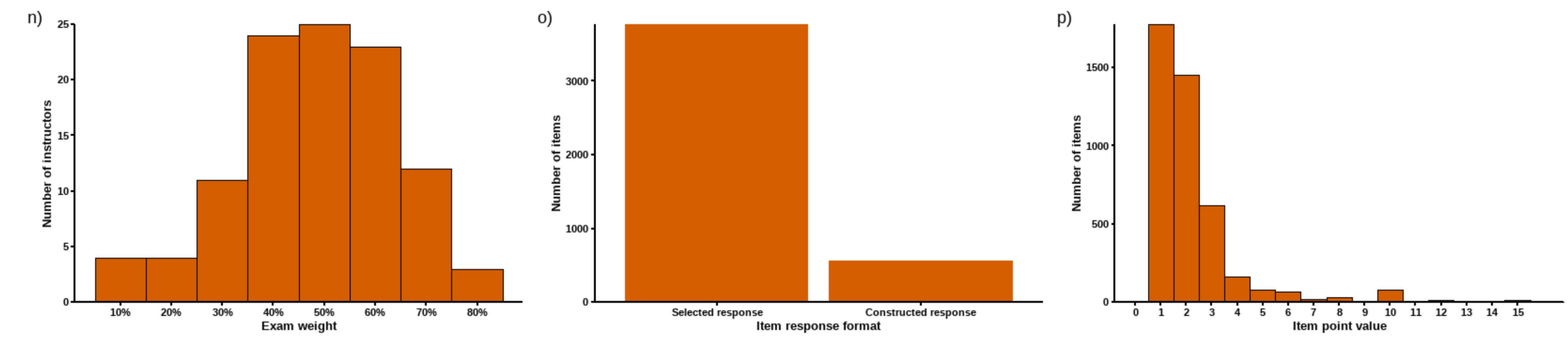

Supplement: S1 Fig — (PDF) [file pone.0312252.s005.pdf]
