# Supplementary material for: Identifying factors associated with instructor implementation of three-dimensional assessment in undergraduate biology courses
Source: PLoS One. 2024 Oct 22;19(10):e0312252. doi: 10.1371/journal.pone.0312252 (PMC11495598; doi:10.1371/journal.pone.0312252)
Supplement: S1 Table — (DOCX) [file pone.0312252.s006.docx]

**Identifying factors associated with instructor implementation of three-dimensional assessment in undergraduate biology courses**

Crystal Uminski, Brian A. Couch

**S1 Table: Applied coding protocol for example item in Fig 2**

| **S1 Table: Applied coding protocol for example item in Fig 2** | | |
| --- | --- | --- |
| **Code** | **Criteria** | **Rationale** |
| Scientific Practice: Developing and Using Models | Student is given or asked to select a mathematical, graphical, computational, symbolic, or pictorial representation and select an appropriate explanation or prediction about an event, observation, or phenomenon based on the representation.   1. Question gives an event, observation, or phenomenon for the student to explain or make a prediction about. 2. Question gives a representation or asks student to select a representation. 3. Question asks student to select an explanation for or prediction about the event, observation, or phenomenon. 4. Question asks student to select the reasoning that links the representation to their explanation or prediction. | 1. Question gives the phenomenon of a DNA replication fork. 2. Question gives a representation of a DNA replication fork. 3. Part A of the question asks student to select how the representation of a DNA replication fork could be modified to be more accurate. 4. Part B of the question asks student to select the reasoning that best explains why the modification selected in Part A would make the representation of a DNA replication fork more accurate. |
| Crosscutting Concept: Structure and Function | To code an assessment task with Structure and Function, the question asks the student to predict or explain a function or property based on a structure, or to describe what structure could lead to a given function or property. | Question asks student to explain the direction of leading and lagging strands based on the structure of the DNA replication fork. |
| Core Idea: Structure and Function | The three-dimensional structure of a molecule and its subcellular localization impact its function, including the ability to catalyze reactions or interact with other molecules. | Question asks student to explain how the structure of leading and lagging strands impact their ability to interact with DNA polymerase. |
