# Supplementary material for: Identifying factors associated with instructor implementation of three-dimensional assessment in undergraduate biology courses
Source: PLoS One. 2024 Oct 22;19(10):e0312252. doi: 10.1371/journal.pone.0312252 (PMC11495598; doi:10.1371/journal.pone.0312252)
Supplement: S2 Table — (DOCX) [file pone.0312252.s007.docx]

**Identifying factors associated with instructor implementation of three-dimensional assessment in undergraduate biology courses**

Crystal Uminski, Brian A. Couch

S2 Table: Institutional Carnegie classifications and geographic regions

| **S2 Table. Institutional Carnegie classifications and geographic regions** | | | | | |
| --- | --- | --- | --- | --- | --- |
| **Institution region** | **Associate’s** | **Baccalaureate** | **Master’s** | **Doctoral** | **Total** |
| Northeast | 4 | 4 | 7 | 6 | 21 |
| Midwest and Great Plains | 6 | 10 | 6 | 7 | 29 |
| Pacific Northwest | 3 | 2 | 0 | 2 | 7 |
| Southeast | 7 | 9 | 4 | 9 | 29 |
| Southwest | 6 | 0 | 2 | 6 | 14 |
| **Total** | 26 | 25 | 19 | 30 | 100 |
| *Note*: Institutional categories are based on Carnegie classifications (Indiana University Center for Postsecondary Research 2021). Institution regions are based on the PULSE regional network classifications (Partnership for Undergraduate Life Sciences Education 2019). | | | | | |
