# Supplementary material for: Identifying factors associated with instructor implementation of three-dimensional assessment in undergraduate biology courses
Source: PLoS One. 2024 Oct 22;19(10):e0312252. doi: 10.1371/journal.pone.0312252 (PMC11495598; doi:10.1371/journal.pone.0312252)
Supplement: S3 Table — (DOCX) [file pone.0312252.s008.docx]

**Identifying factors associated with instructor implementation of three-dimensional assessment in undergraduate biology courses**

Crystal Uminski, Brian A. Couch

S3 Table: Self-reported demographic information of undergraduate biology instructors

| S3 Table. Self-reported demographic information of undergraduate biology instructors | | |
| --- | --- | --- |
| *Characteristic* | n | % |
| Gender^a^ | | |
| Female | 67 | 60 |
| Male | 42 | 38 |
| Self-described | 0 | 0 |
| Preferred not to disclose | 2 | 2 |
| Race/ethnicity^b^ | | |
| Non-underrepresented | 97 | 87 |
| Underrepresented | 11 | 10 |
| Self-described | 1 | 1 |
| Preferred not to disclose | 2 | 2 |
| Teaching experience as an instructor of record | | |
| 0-1 year | 5 | 5 |
| 2-5 years | 20 | 18 |
| 6-10 years | 30 | 27 |
| 11-15 years | 29 | 26 |
| 16-20 years | 10 | 9 |
| 21-25 years | 11 | 10 |
| > 25 years | 6 | 5 |
| ^a^This was the original terminology included in the survey, but we note that the categories here are more representative of sex rather than gender based on current guidelines (American Psychological Association, 2022).  ^b^We use the term “underrepresented” here to convey our focus on racial/ethnic groups that have faced disproportionate challenges within STEM disciplines, including Black/African American, Hispanic/Latinx, American Indian/Alaska Native, and Native Hawaiian/Pacific Islander. This grouping is not intended to obscure the unique histories and identities of any group. | | |
