# Supplementary material for: Identifying factors associated with instructor implementation of three-dimensional assessment in undergraduate biology courses
Source: PLoS One. 2024 Oct 22;19(10):e0312252. doi: 10.1371/journal.pone.0312252 (PMC11495598; doi:10.1371/journal.pone.0312252)
Supplement: S4 Table — (DOCX) [file pone.0312252.s009.docx]

**Identifying factors associated with instructor implementation of three-dimensional assessment in undergraduate biology courses**

Crystal Uminski, Brian A. Couch

S4 Table: Categories of lower-division biology courses included in the sample

| **S4 Table. Categories of lower-division biology courses included in the sample** | | |
| --- | --- | --- |
| ***Course category***^a^ | **n** | **%** |
| Introductory – Cell/Molecular | 32 | 29 |
| Introductory – Organismal | 31 | 28 |
| Introductory – General Biology | 26 | 23 |
| Ecology/Evolution | 6 | 5 |
| Genetics | 3 | 3 |
| Microbiology | 3 | 3 |
| Anatomy/Physiology | 3 | 3 |
| Cell/Molecular Biology | 2 | 2 |
| Environmental Science | 2 | 2 |
| Plant Biology | 2 | 2 |
| Zoology | 1 | < 1 |
| ***Lab courses*** |  |  |
| Course has an associated lab component | 95 | 86 |
| Course does not have an associated lab component | 16 | 14 |
| ^a^If course category was not evident based on the title of the course, we used the content in the course syllabus to designate the categories. We categorized introductory-series courses that primarily deal with molecules, cells, and genetics as “Introductory – Cell/Molecular,” introductory-level courses that primarily deal with animal systems, biodiversity, ecology, and evolution topics as “Introductory – Organismal,” and courses that broadly span both cell/molecular biology and ecology/evolution topics as “Introductory – General Biology.” | | |
