# Supplementary material for: Identifying factors associated with instructor implementation of three-dimensional assessment in undergraduate biology courses
Source: PLoS One. 2024 Oct 22;19(10):e0312252. doi: 10.1371/journal.pone.0312252 (PMC11495598; doi:10.1371/journal.pone.0312252)
Supplement: S5 Table — (DOCX) [file pone.0312252.s010.docx]

**Identifying factors associated with instructor implementation of three-dimensional assessment in undergraduate biology courses**

Crystal Uminski, Brian A. Couch

S5 Table: Descriptions of item types

| **S5 Table: Descriptions of item types** | |
| --- | --- |
| **Item type** | **Description** |
| *Selected-response* | |
| Multiple-choice | The test-taker selects one option from a list of two or more provided options. |
| Multiple-select | A multiple-choice item where more than one option can be selected as correct. |
| True-false | The test-takers select whether a single statement is true or false. Unlike multiple-true-false, there is no preceding prompt or question linking multiple statements together. |
| Multiple-true-false | A form of multiple select where the options consist of binary factual statements and are preceded by a prompt or question statement linking the options together. |
| Matching | For each option in one list, the test-taker selects the correct match from a second list. Matching options may be presented as a series of items where each item in the series has the same set of common options. |
| Reorder | Test-takers put a series of provided options into a sequence or specified order. |
| *Constructed-response* | |
| Fill-in-the-blank | Test-takers fill in a word or a short phrase that is missing from the stimulus and there is not a list of responses (i.e., a “word bank”) provided. |
| Short answer | Test-takers respond to the item with a word, phrase, or response that does not exceed one paragraph (approximately 3-4 sentences). |
| Essay | Test-taker respond to an essay item that typically requires more than one paragraph. Essay items often use verbs such as “explain” or “justify” to elicit longer responses from test-takers. |
| Cluster | Test-takers respond to a series of items that share a common stimulus. The series of items are designed as sub-parts or sub-items, which may or may not be scored independently. Cluster items often differ from essay items in that test-takers are provided a bulleted or numbered list of discrete sub-parts to respond to rather than a single paragraph of text directions. |
| Math manipulation | Test-takers manipulate information to solve mathematical or algorithmic problems. |
| Modeling | Test-takers respond to the item by creating a model of a biological phenomenon or by adding to, contributing to, or otherwise modifying an existing model. |
| Discipline-specific | Test-takers use procedures, algorithms, or other processes that are specific to biological sciences but are not easily categorized as strictly modeling or mathematical manipulation of information. Examples include manipulating genetic sequences or completing Punnett squares. |
