# Supplementary material for: Identifying factors associated with instructor implementation of three-dimensional assessment in undergraduate biology courses
Source: PLoS One. 2024 Oct 22;19(10):e0312252. doi: 10.1371/journal.pone.0312252 (PMC11495598; doi:10.1371/journal.pone.0312252)
Supplement: S6 Table — (DOCX) [file pone.0312252.s011.docx]

**Identifying factors associated with instructor implementation of three-dimensional assessment in undergraduate biology courses**

Crystal Uminski, Brian A. Couch

S6 Table: Tukey post hoc contrasts comparing between the probability of three-dimensional alignment based on institution type

| **S6 Table: Tukey post hoc contrasts comparing between the probability of three-dimensional alignment based on institution type** | | | | |
| --- | --- | --- | --- | --- |
| **Contrast** | **Estimate** | **SE** | ***z*** | ***p*** |
| Baccalaureate – Associate’s | -0.80 | 0.50 | -1.58 | 0.39 |
| Master’s – Associate’s | -0.22 | 0.53 | -0.41 | 0.98 |
| Doctoral – Associate’s | 0.68 | 0.47 | 1.44 | 0.47 |
| Master’s – Baccalaureate | 0.58 | 0.53 | 1.10 | 0.69 |
| Doctoral – Baccalaureate | 1.48 | 0.47 | 3.18 | *<0.001 |
| Doctoral – Master’s | 0.89 | 0.50 | 1.78 | 0.28 |
