# Supplementary material for: Identifying factors associated with instructor implementation of three-dimensional assessment in undergraduate biology courses
Source: PLoS One. 2024 Oct 22;19(10):e0312252. doi: 10.1371/journal.pone.0312252 (PMC11495598; doi:10.1371/journal.pone.0312252)
Supplement: S7 Table — (DOCX) [file pone.0312252.s012.docx]

**Identifying factors associated with instructor implementation of three-dimensional assessment in undergraduate biology courses**

Crystal Uminski, Brian A. Couch

**S7 Table: Item types of three-dimensional and non-three-dimensional items**

| **S7 Table. Item types of three-dimensional and non-three-dimensional items** | | | | | |
| --- | --- | --- | --- | --- | --- |
| **Item Type** | | **Total** | **Non-3D** | **3D** | **Percent 3D** |
| **Selected-response** | | 3771 | 3665 | 106 | 2.8 |
|  | Multiple-choice | 3240 | 3145 | 95 | 2.9 |
|  | Matching | 250 | 240 | 10 | 4.0 |
|  | True-false | 173 | 173 | 0 | 0.0 |
|  | Multiple-select | 65 | 64 | 1 | 1.5 |
|  | Multiple-true-false | 30 | 30 | 0 | 0.0 |
|  | Reorder | 13 | 13 | 0 | 0.0 |
| **Constructed-response** | | 566 | 436 | 130 | 23.0 |
|  | Short answer | 287 | 216 | 71 | 24.7 |
|  | Fill-in-the-blank | 101 | 100 | 1 | 1.0 |
|  | Cluster | 76 | 41 | 35 | 46.1 |
|  | Essay | 36 | 20 | 16 | 44.4 |
|  | Model | 35 | 29 | 6 | 17.1 |
|  | Discipline-specific | 16 | 15 | 1 | 6.3 |
|  | Math manipulation | 15 | 15 | 0 | 0.0 |
